# Modeling immune responses to autologous and allogeneic human stem cell–derived islet grafts in vivo

**DOI:** 10.1172/jci.insight.200738

**Published:** 2026-06-08

**Authors:** Camillo Bechi Genzano, Giorgia Zanetti, Qian Du, Daniel Traum, Deeksha Lahori, Grant M. Downes, Sakshi A. Bhatele, Xiaolan Ding, Kyle D. Apley, Rebuma Firdessa Fite, Matthew Ishahak, Enrique Eduardo Sanchez-Castro, Jeffrey R. Millman, Yiming Luo, Klaus H. Kaestner, Cory Berkland, Dieter Egli, Megan Sykes, Remi J. Creusot

**Affiliations:** 1 Columbia Center for Translational Immunology and Department of Medicine,; 2 Naomi Berrie Diabetes Center, and; 3 Department of Pediatrics and Columbia Stem Cell Initiative, Columbia University Medical Center, New York, New York, USA.; 4 Department of Genetics and Institute for Diabetes, Obesity and Metabolism, University of Pennsylvania, Philadelphia, Pennsylvania, USA.; 5 Bioengineering Graduate Program, University of Kansas, Lawrence, Kansas, USA.; 6 Department of Biomedical Engineering and; 7 Division of Endocrinology, Metabolism and Lipid Research, Washington University School of Medicine, St. Louis, Missouri, USA.; 8 Division of Rheumatology and Clinical Immunology, Department of Medicine, Columbia University Medical Center, New York, New York, USA.; 9 Departments of Biomedical Engineering and Chemistry, Washington University in St. Louis, St. Louis, Missouri, USA.; 10 Departments of Surgery and of Microbiology & Immunology, Columbia University Medical Center, New York, New York, USA.

**Keywords:** Autoimmunity, Endocrinology, Immunology, Beta cells, Human stem cells, T cells

## Abstract

Stem cell–derived β cells offer a promising approach for type 1 diabetes (T1D) treatment. However, the processes of graft infiltration and rejection by immune cells remain poorly understood in humans. In this study, autologous or allogeneic stem cell–derived islets (SC-islets) were transplanted in human immune system mice and analyzed 14 to 18 weeks later. Imaging mass cytometry revealed unique characteristics of SC-islet grafts, including a high percentage of glucagon^+^ cells and the presence of cysts and CD57^+^ enterochromaffin cells, features not typically observed in endogenous or transplanted allogeneic primary pancreatic islets. Allogeneic SC-islet grafts exhibited heavy immune infiltration, cell proliferation, and pro-fibrotic processes, whereas autologous grafts showed minimal infiltration and little fibrosis. In some mice, autologous T cells expressing islet antigen-reactive (IAR) T cell receptors (TCRs) were adoptively transferred. Three weeks after transfer, autologous grafts injected with IAR-TCR^+^ T cells showed negligible immune infiltration, even though IAR-TCR^+^ T cells were detected in the spleen. Under the conditions tested, human SC-islet grafts were not rejected by an autologous immune system, even in the presence of autoreactive T cells, pointing to several limitations that remain to be addressed for a model of spontaneous autologous SC-islet infiltration and destruction.

## Abstract 2

Our study shows how the human immune system responds to transplanted stem cell-derived insulin-producing cells, providing a powerful model to improve future T1D therapies.

## Introduction

Despite advances in insulin therapy, type 1 diabetes (T1D) management with exogenous insulin remains a major challenge because imprecise glycemic regulation leaves patients at risk of developing long-term complications (1). Islet transplantation offers better prospects through tighter glycemic control, but donors are scarce, and islets are costly to isolate and transplant. Moreover, HLA mismatch requires the recipient to remain under lifelong immunosuppressive treatments, which is often found to be unjustifiable given the risks (2). Recently, primary islets were partially gene edited to be “hypoimmune” (able to evade immune detection) and to prevent their rejection in a patient with T1D (3), thereby avoiding general immunosuppression.

To address the limited supply of primary islets from deceased donors, stem cell–derived islets (SC-islets) hold great promise (4, 5). They not only provide a potential solution for replacing β cells lost in patients but also enable the generation of autologous (patient-specific) β cells, thereby avoiding alloimmune rejection (6, 7). SC-islets — whether allogeneic (from a human donor) or autologous (from the patient) — both serve as viable options for transplantation, although allogeneic islets require immunosuppressive treatment to prevent rejection (8). Advancements in hypoimmune cell strategies have also been applied to SC-islets for the purpose of using allogeneic cells as off-the-shelf products (9, 10). On the other hand, autologous islet transplants are expected to require protection only from autoreactive islet antigen-reactive (IAR) T cells. Given that clinical trials of SC-islet transplantation have begun only recently (11), it is not yet clear whether these grafts elicit the same immune response as primary islets isolated from human pancreata. Moreover, because nonencapsulated islet grafts are typically not retrievable, the mechanisms underlying immune cell infiltration and rejection in these new types of grafts will remain poorly understood unless adequate models are developed to recapitulate these processes for human cells. Such in vivo models would be useful to evaluate drugs and therapies for graft rejection.

Using human immune system (HIS) mice (12, 13), we set out to investigate features and outcomes of autologous and allogeneic human SC-islet grafts exposed to a reconstituted immune system in vivo. Additionally, we introduced multiple HLA class I– and class II–restricted islet antigen-reactive T cell receptors (IAR-TCRs) into T cells to increase the frequency of autoreactive T cells. With this approach, we attempted to visualize spontaneous islet infiltration by these T cells without the use of peptide immunization or β cell damage, both of which were previously employed to stimulate infiltration of pancreatic islets in another HIS mouse model (14).

## Results

### Experimental design.

**Figure 1:**
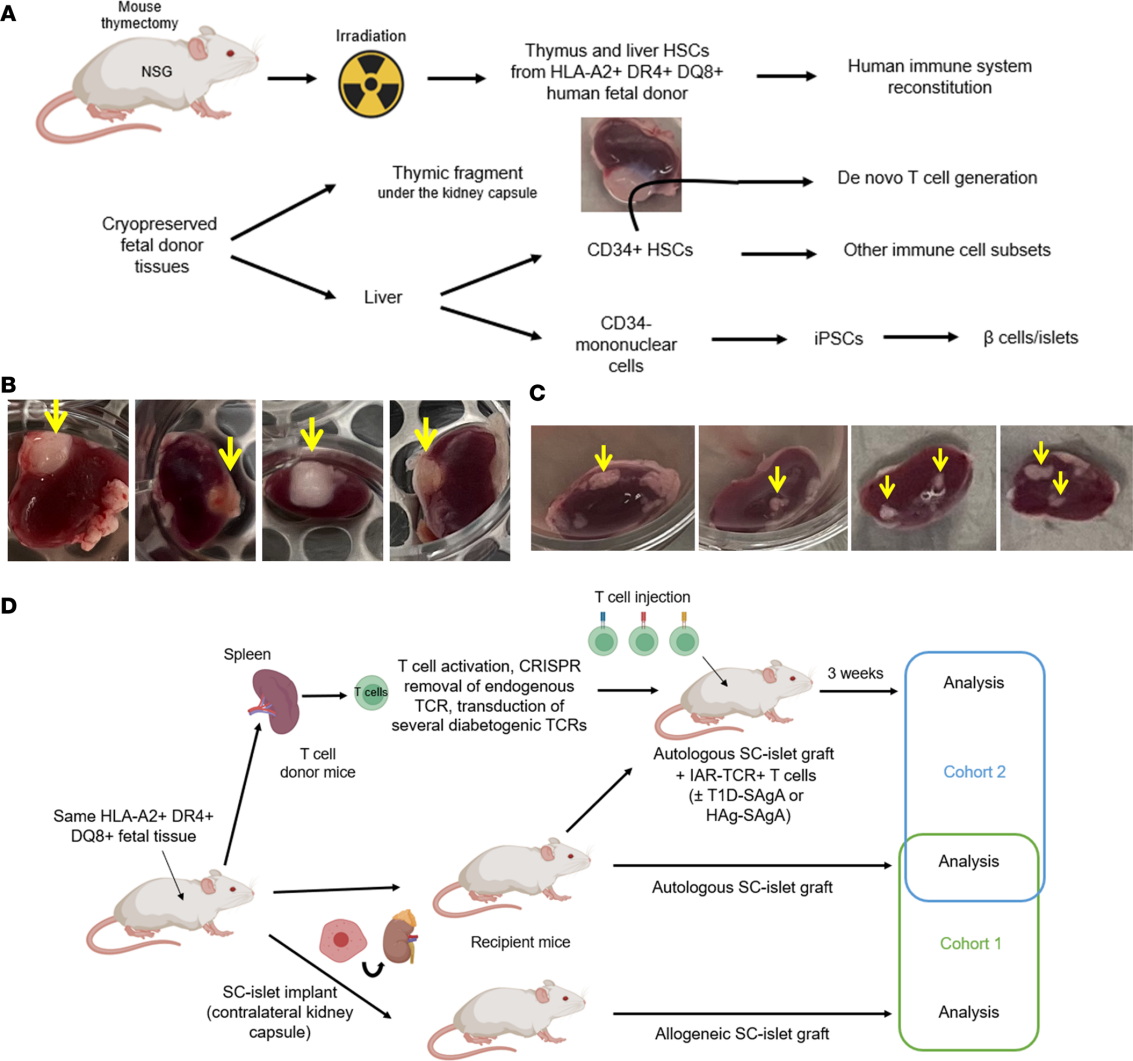
Experimental design. (**A**) HIS mouse generation using transplanted human fetal liver CD34^+^ cells and thymic tissue with HLA-A2, HLA-DR4, and HLA-DQ8 alleles. Either autologous (from iPSCs produced from CD34^–^ liver cells of the same human fetal donor) or allogeneic (fully HLA-mismatched) SC-islets were grafted under the kidney capsule. (**B** and **C**) Examples of thymic (**B**) and SC-islet grafts (**C**) collected during graft retrieval, 20 and 15 weeks after implantation, respectively. (**D**) Schematic representation of experimental groups and how they were generated.

We generated HIS mice by transplanting human fetal liver CD34^+^ cells and thymic tissue with HLA-A2, HLA-DR4, and HLA-DQ8 alleles in thymectomized NOD-SCID gamma (NSG) mice (Figure 1A and Supplemental Table 1; supplemental material available online with this article; https://doi.org/10.1172/jci.insight.200738DS1). Fetal liver CD34^–^ cells were used to generate autologous induced pluripotent stem cells (iPSCs), which were then differentiated into SC-islets. In all mice, fetal thymic tissue pieces were implanted under the right kidney capsule (examples in Figure 1B), and SC-islets were later grafted under the left kidney capsule (examples in Figure 1C). We generated 2 experimental cohorts with the same donor tissues and compared autologous (mice generated in cohorts 1 and 2) and fully HLA-mismatched allogeneic (mice generated in cohort 1) SC-islets. We also assessed the infiltration of autologous IAR T cells (mice generated in cohorts 1 and 2; Figure 1D) in a subgroup of mice grafted with autologous SC-islets.

### HIS reconstitution and iPSC-derived islet generation.

**Figure 2:**
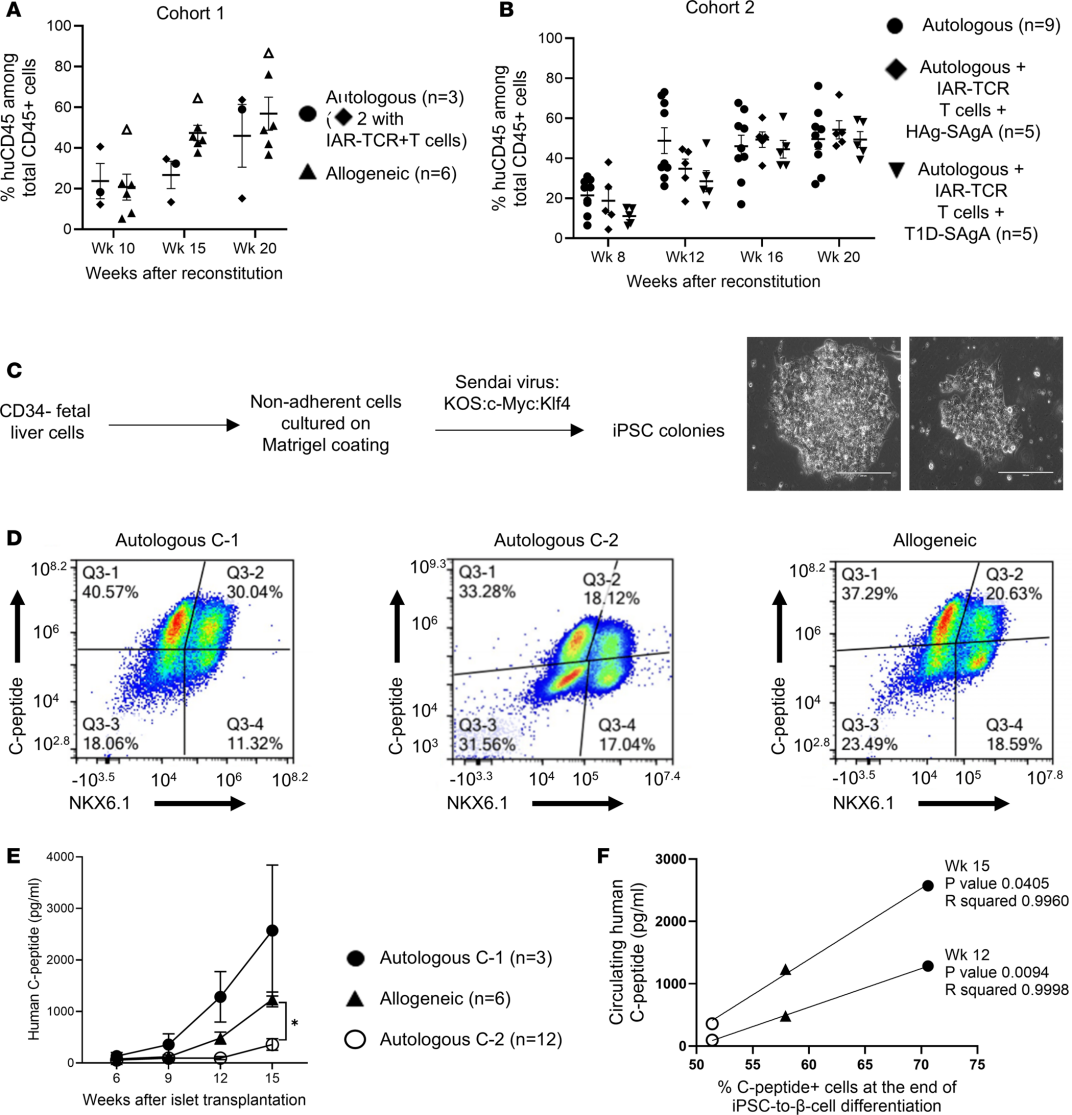
Human immune system and stem cell–derived islet reconstitution. (**A** and **B**) Human immune system reconstitution in cohort 1 (**A**) and 2 (**B**); human chimerism is measured as the percentage of human CD45^+^ cells among total CD45^+^ cells. Each symbol represents an individual animal. In **A**, a hollow triangle represents an allogeneic mouse not injected with polyclonal T cells. (**C**) CD34^–^ nonadherent fetal liver cells reprogramming into iPSCs using Klf4, Oct3/4, Sox2, and c-Myc transcription factors delivered via the nonintegrating Sendai virus, with examples of iPSC colonies. Scale bar: 200 µm. (**D**) iPSC-to-β cell differentiations performed in the study. (**E**) Circulating C-peptide levels in autologous (cohort 1 and 2) and allogeneic grafts. In autologous cohort 1 and 2, *n* = 2/3 and *n* = 3/12 mice were injected with IAR-TCR^+^ T cells that did not infiltrate the grafts, respectively, and *n* = 5/6 allogeneic mice were injected with additional in vitro activated polyclonal T cells from a mouse with the same HIS. In **E**, the asterisk indicates the level of significance (*P < 0.05) when comparing the percentage of circulating C-peptide in allogeneic and autologous cohort 2 at weeks 12 and 15 after β cell transplantation. (**F**) Correlation between percentage of C-peptide^+^ cells in islet before transplant and circulating human C-peptide levels 12 and 15 weeks later. In **A**, **B**, and **E**, data show the mean ± SEM. In **F**, different groups are represented with the same symbols as **E,** and each symbol represents the mean of the group. Statistical analysis was performed using a mixed-effects model with Geisser-Greenhouse correction and Tukey’s post hoc test for **E** and a simple linear regression for **F**. C-1, cohort 1; C-2, cohort 2.

We observed progressive reconstitution of the HIS, as previously described (14–16). Mice developed high levels of human chimerism (Figure 2, A and B) and T cells (Supplemental Figure 1, A and B), indicating successful engraftment of the human thymus. The reconstitution of circulating B cells and monocytes is indicated in Supplemental Figure 1, A and B. Of note, the NSG mice used in our experiments do not support robust development of NK cells, possible contributors to allorejection (13, 17).

To achieve a system in which the SC-islets are isogenic to the immune system, CD34^–^ fetal liver cells from the same human fetal donor were reprogrammed to iPSCs (Figure 2C) using Klf4, Oct3/4, Sox2, and c-Myc transcription factors delivered by the nonintegrating Sendai virus (18). We tested the reprogramming of both fibroblastic-like fetal liver cells and nonadherent fetal liver cells, cultured either on a Matrigel coating or on feeder cells (mouse embryonic fibroblasts). Nonadherent cells, cultured on a Matrigel coating, produced iPSC colonies with high efficiency (Figure 2C) and were then differentiated into SC-islets, following previously described protocols to prevent teratoma formation (19, 20). For the allogeneic islets, iPSCs previously derived from reprogrammed skin fibroblasts were used (20). At the end of all 3 differentiations performed in the study, at least 50% of the cells expressed C-peptide, although at varying percentages (Figure 2D, 71%, 51%, and 58% for autologous cohort 1, autologous cohort 2, and allogeneic, respectively). In addition, 35%–45% of the C-peptide^+^ cells expressed NKX6.1. We measured circulating levels of human C-peptide in the serum to confirm the relative survival and function of the grafts (Figure 2E). Grafts of both types started producing levels of human C-peptide above background (>10 pg/mL) 6 weeks after implantation, and levels increased over time. Circulating human C-peptide levels correlated with the efficiency of iPSC-to-β cell differentiation, as indicated by the percentage of C-peptide^+^ cells prior to transplantation (Figure 2F).

### Autologous versus allogeneic iPSC-derived islet graft immune infiltration.

**Figure 3:**
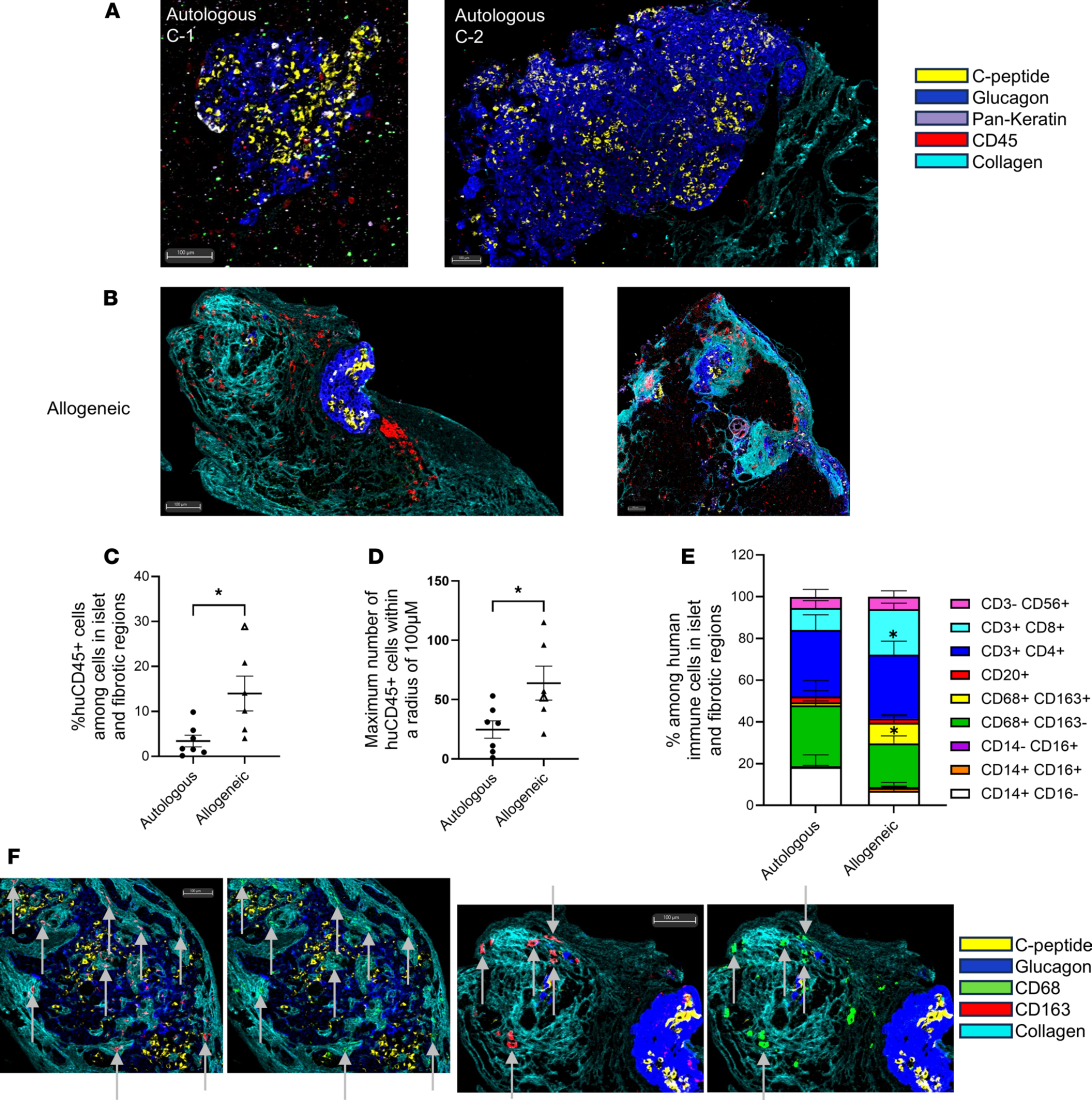
Autologous and allogeneic stem cell–derived islet graft human immune infiltration. (**A**) Representative images of autologous SC-islet graft human immune infiltration. Scale bar: 100 μm. (**B**) Representative images of allogeneic SC-islet graft human immune infiltration, without (left) or with (right) injection of additional in vitro activated polyclonal T cells. Scale bar: 100 μm. (**C**) Quantification of CD45^+^ cell infiltration from IMC data. (**D**) Relative clustering, based on maximum number of CD45^+^ cells within a radius of 100 μm in autologous and allogeneic grafts. (**E**) Composition of immune infiltrates in autologous and allogeneic grafts. (**F**) Representative images of CD68^+^ CD163^+^ macrophage infiltration (indicated with arrows) in allogeneic grafts. The rightmost panel in **F** is a magnified view of the area shown in the left panel in **B**, with some markers changed. Markers used for analysis are indicated on the right. Scale bar: 100 μm. In the experiments, *n* = 5/6 allogeneic mice were injected with additional in vitro activated polyclonal T cells; autologous mice did not receive additional in vitro activated polyclonal T cells (hollow triangle represents the allogeneic mouse not injected with polyclonal T cells). In **C** and **D**, each symbol represents 1 animal. Data shown as mean ± SEM; statistical analysis was performed using an unpaired 2-tailed *t* test for **C**–**E**. *P < 0.05. C-1, cohort 1; C-2, cohort 2.

We assessed SC-islet graft characteristics using imaging mass cytometry (IMC) (21). As expected, allogeneic grafts displayed significantly greater immune infiltration (CD45^+^ cells) than autologous grafts (Figure 3, A–C, and Supplemental Figure 2, A and B). Although collagen staining suggested greater fibrosis in allografts (Figure 3, A and B), accurate quantification was limited by difficulty distinguishing disease-associated collagen deposition from collagen staining of the renal capsule or any scarring caused by the islet implantation procedure. Allogeneic graft infiltrates were characterized by increased CD45^+^ cell clustering (Figure 3, B and D). In autologous grafts, any CD45^+^ cell clustering was typically observed around cyst-like structures expressing the ductal marker cytokeratin 19 (CK19) (examples in Supplemental Figure 2C) (19).

Although CD45^+^ cells were more abundant as a fraction of total cells in allografts than in autografts (Figure 3C), the immune cell composition was similar between the 2 types of graft, except for CD163^+^ CD68^+^ macrophages and CD8^+^ T cells, which were significantly enriched in islet and fibrotic regions of allogeneic grafts (Figure 3E). Of note, CD68^+^ CD163^+^ macrophages (Figure 3F) represent a cell population involved in antiinflammatory responses and tissue remodeling and have been shown to contribute to allograft fibrosis (22), whereas CD8^+^ T cells (Supplemental Figure 3A) are among the primary mediators of allorejection (23). In some allogeneic samples (*n* = 3/6), we identified active destruction and remodeling with the presence of FoxP3^+^ and Granzyme B^+^ T cells (Supplemental Figure 3B).

### Undetectable IAR-TCR^+^ T cell infiltration in autologous iPSC-derived islet grafts.

**Figure 4:**
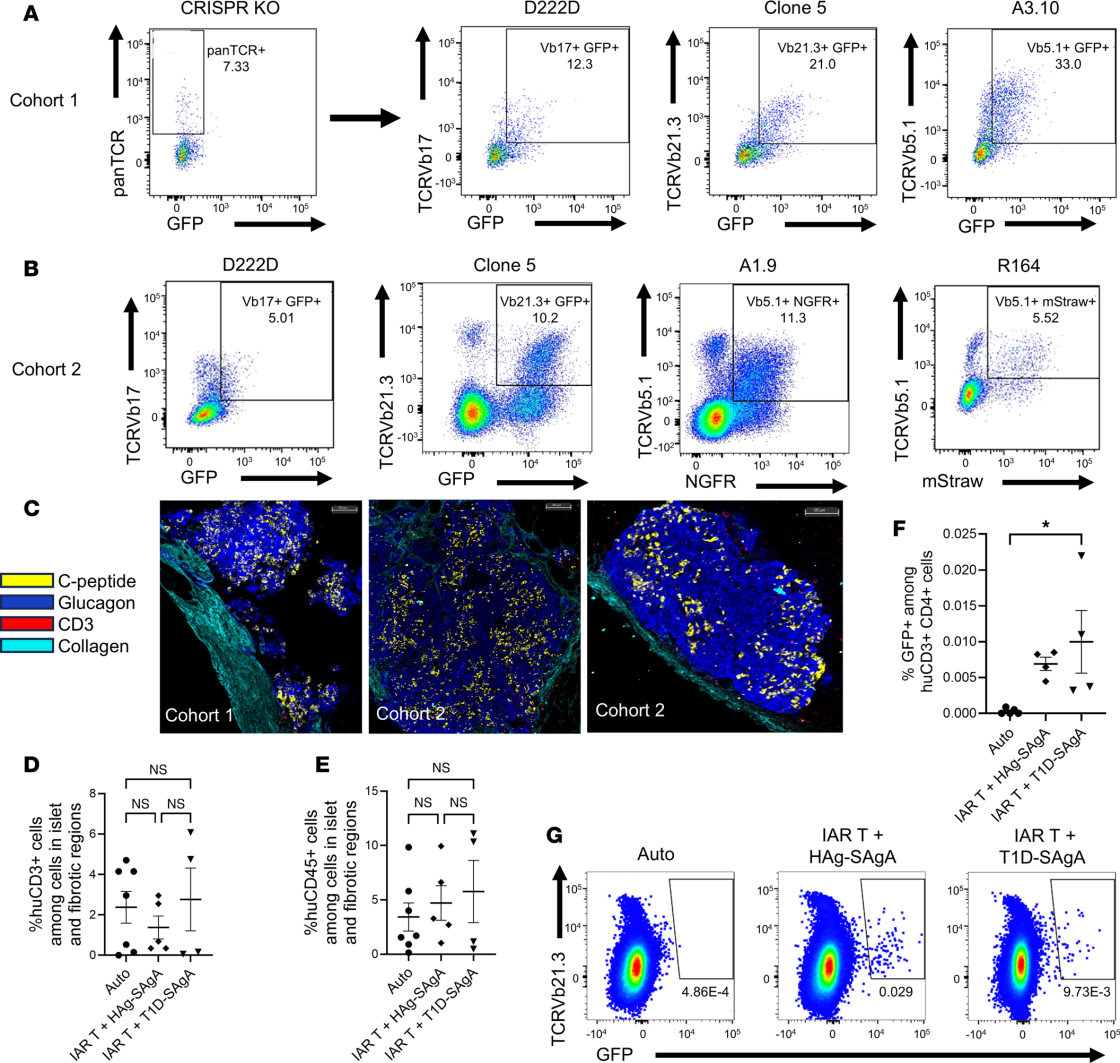
Undetectable infiltration by autologous IAR-T cells. (**A** and **B**) Autologous T cell transduction with IAR-TCR–encoding lentiviruses performed in experiment 1 (**A**) and 2 (**B**). (**C**) Examples of autologous grafts injected with IAR-TCR^+^ T cells without T1D-SAgA. Scale bar: 100 µm. (**D** and **E**) Quantification of CD3^+^ (**D**) and CD45^+^ (**E**) cell graft infiltration from IMC data. (**F**) Identification of Clone 5 T cells in the spleen 3 weeks after adoptive transfer. *P < 0.05. (**G**) Quantification of Clone 5 T cells in the spleen. For **C**, markers used for analysis are indicated on the left of the images. Data shown as the mean ± SEM; statistical analysis was performed using 1-way ANOVA/Tukey’s post hoc test for **D**–**F**.

In both cohort 1 (*n* = 2/3 mice) and cohort 2 (*n* = 10/16 mice), we adoptively transferred autologous HIS mouse–derived T cells transduced to express IAR-TCRs in mice implanted with autologous grafts to model and study autoimmune responses (Figure 1D). The T cell clones used are listed in Table 1 and were previously identified in the islets or blood of patients with T1D (14, 24–26). We isolated splenocytes from a donor mouse of the same cohort and activated human T cells with human anti-CD3/CD28/CD2. Activated T cells were transduced to express IAR-TCRs (with or without CRISPR-mediated ablation of the endogenous TCR) (Figure 4, A and B). Reactivity of the Clone 5 TCR was validated ex vivo by restimulation with peptide-pulsed K562-DQ8 cells of transduced splenocytes containing detectable Vβ21.3^+^ GFP^+^ CD4^+^ T cells (Supplemental Figure 4, A–D), while other TCR clones (D222D, A1.9, A3.10, R164) were validated in vitro using Jurkat T cells (unpublished observations). After a 2-week expansion in vitro, T cells displayed an effector memory-TEMRA phenotype (Supplemental Figure 4E). We adoptively transferred 0.2 × 10^6^ to 0.5 × 10^6^ TCR^+^ reporter^+^ cells per clone into each recipient mouse, corresponding to a combined 1 × 10^6^ to 2 × 10^6^ IAR-TCR^+^ T cells per mouse. In total, 6 × 10^6^ (cohort 1) and 18 × 10^6^ (cohort 2) activated T cells — comprising both IAR-TCR^+^ and polyclonal T cells — were transferred. We previously reported that peptides corresponding to T1D antigen epitopes, when delivered in the form of soluble antigen arrays (SAgAs), were able to reprogram antigen-specific T cells and attenuate diabetes development in nonobese diabetic mice (27, 28). We took advantage of these HIS mice to perform an exploratory assessment of differences in the frequency and phenotype of human IAR-TCR^+^ T cells in response to relevant and irrelevant SAgAs. Thus, all mice from cohort 2 were also subsequently treated with SAgAs carrying either T1D-relevant antigens (human proinsulin (29, 30) and InsB_9-23_ peptides (31), hereafter referred to as T1D-SAgA) or an irrelevant control antigen (HAg_306-318_ peptide from influenza HA, HAg-SAgA) to evaluate whether these IAR-TCR^+^ T cells could be targeted and modulated by an antigen-specific therapy.

Three weeks after transfer, autologous grafts injected with IAR-TCR^+^ T cells appeared similar to non-transferred autologous grafts, with no significant increase of CD3^+^ T cells (Figure 4, C and D) or CD45^+^ cells (Figure 4E), irrespective of IAR-TCR^+^ T cell injection and of the treatment (HAg-SAgA versus T1D-SAgA). There were no significant alterations in HLA class I expression that could have explained the lack of graft infiltration (Supplemental Figure 4F). Clone 5 T cells, specific for InsB_9-23_, were detected in the spleen of all mice injected with IAR-TCR^+^ T cells 3 weeks after transfer (Figure 4, F and G, and Supplemental Figure 5, A and B). A3.10-TCR T cells, only injected in cohort 1, were also found in the spleen (Supplemental Figure 5, A and B), while other T cell clones were not detected (data not shown).

In SAgA-treated mice, human circulating C-peptide could not be used as a reliable measure of human β cell function, because the presence of Proinsulin-SAgA in the serum contributed to a high background detection (detection of C-peptide within Proinsulin-SAgAs, Supplemental Figure 5D). However, Clone 5 TCR^+^ CD4^+^ T cells detected in the spleen exhibited differences in their activation profile with downregulation of CD25 and upregulation of PD-1 after T1D-SAgA treatment relative to control HAg-SAgA treatment (Supplemental Figure 6, A–C).

### Composition and function of iPSC-derived islet grafts.

**Figure 5:**
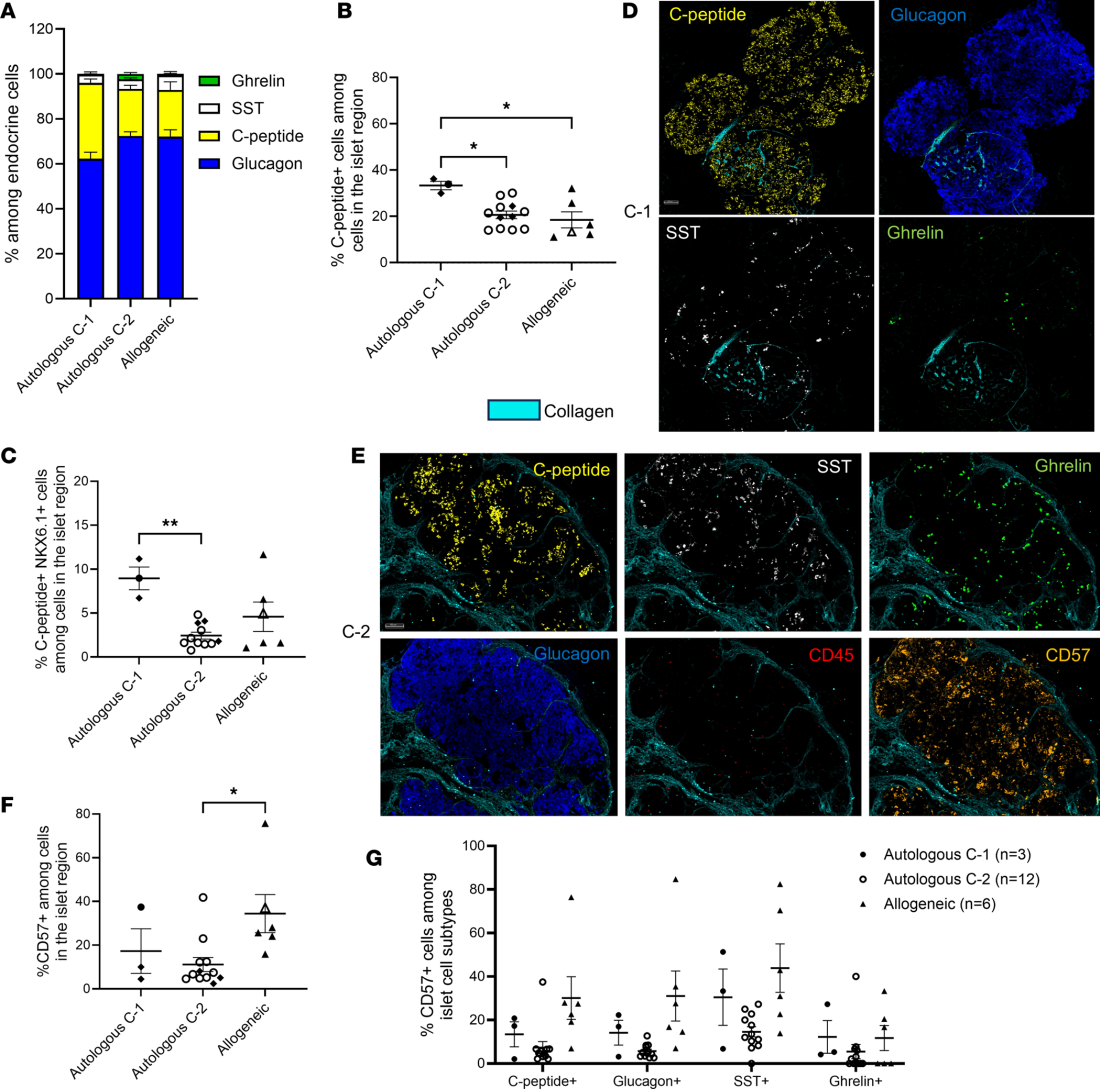
Endocrine composition of grafts. (**A**) Endocrine cell populations found in the grafts, represented as percentage of total endocrine cells. (**B** and **C**) Comparison of C-peptide^+^ (**B**) and C-peptide^+^ NKX6.1^+^ cells (**C**) among cells found in the islet region between autologous (cohort 1, cohort 2) and allogeneic grafts. (**D**) Representative images of endocrine cell subpopulations in autologous grafts from cohort 1. Scale bar: 100 µm. (**E**) Representative images of endocrine cell subpopulations and CD57 expression in the grafts of autologous cohort 2. Scale bar: 100 µm. (**F**) Quantification of CD57^+^ cells in the grafts. (**G**) Quantification of CD57 expression among SC-islet cell subtypes. For **D** and **E**, markers used for analysis are indicated in the figure. In autologous cohorts 1 and 2, *n* = 2 and *n* = 3 mice were injected with IAR-TCR^+^ T cells, respectively; *n* = 5 allogeneic mice were injected with additional in vitro activated polyclonal T cells (a hollow triangle represents the allogeneic mouse not injected with additional polyclonal T cells, and autologous mice injected with IAR-TCR^+^ T cells are represented as solid circles). Mean ± SEM; 1-way ANOVA/Tukey’s post hoc test was used for **B**, **C**, and **F**. *P < 0.05, **P < 0.01. C-1, cohort 1; C-2, cohort 2; SST, somatostatin.

Major islet cell subtypes could be identified in both autologous and allogeneic grafts (Figure 5A), with a prevalence of glucagon^+^ cells in all grafts. Consistent with C-peptide levels in the serum (Figure 2E), the percentage of C-peptide^+^ cells was higher in grafts from autologous cohort 1 (Figure 5B), and the percentage of C-peptide^+^ NKX6.1^+^ cells (Figure 5C and Supplemental Figure 7A) reflected the efficiency of iPSC-to-β cell differentiation in vitro, with the highest expression in autologous cohort 1 and the lowest in autologous cohort 2 (Figure 2D). Other endocrine cell subpopulations, such as somatostatin^+^ or ghrelin^+^ cells, were also observed but at low frequency (Figure 5, A, D, and E).

CD57 was expressed by a substantial fraction of cells in all grafts, which, unexpectedly, were not CD45^+^ but positive for endocrine markers (Figure 5E, Supplemental Figure 7, B and C). Allogeneic grafts had a higher frequency of CD57^+^ cells (Figure 5F). We observed CD57 expression among a minority of cells in all islet cell subtypes (Figure 5G), with the second autologous SC-islet differentiation showing the lowest percentage. Analysis of previously published single-cell RNA-Seq (scRNA-Seq) data (32–39), corresponding to other SC-islet differentiations, indicated that *CD57* expression gradually increases during the differentiation process (Supplemental Figure 7D). The highest *CD57* expression was seen in enterochromaffin cells, an off-target cell type generated by current in vitro differentiation protocols and identified based on *TPH1* expression (Supplemental Figure 7E and Supplemental Figure 8). As in our samples (protein expression; Figure 5G), *CD57* expression was also detectable in α, β, and δ cells (mRNA expression; Supplemental Figure 7E and Supplemental Figure 8B). Cells that expressed CD57 in the absence of islet endocrine or immune markers (possibly residual enterochromaffin cells) represented no more than 15% of all CD57^+^ cells.

Although no teratoma formation was observed, the aforementioned cyst-like structures composed of CK19-expressing cells were present in some grafts (*n* = 8/21; Supplemental Figure 2C for example) and are considered to also be a by-product of current iPSC-to-β cell differentiation protocols (19).

Thorngren and colleagues have recently reported the absence of human endothelial CD31^+^ cells in SC-islets, with graft vascularization exclusively dependent on recipient (murine) blood vessels (40). Likewise, we confirmed the absence of human CD31 in all the grafts analyzed by IMC (data not shown).

### More active proliferation in allogeneic compared with autologous grafts.

**Figure 6:**
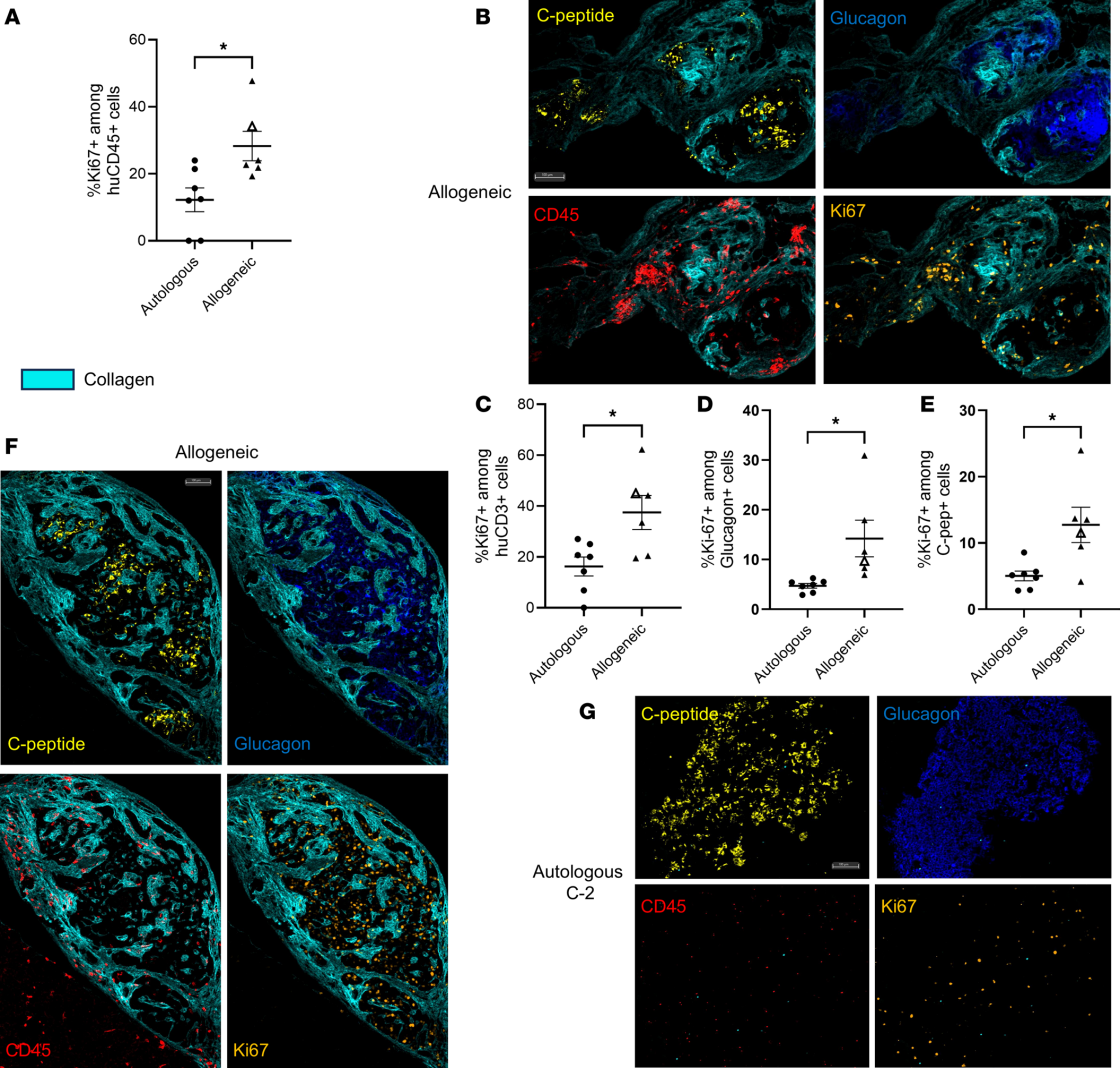
Proliferation in allogeneic grafts based on Ki67 expression. (**A**) Quantification of CD45^+^ cell proliferation in autologous and allogeneic grafts. (**B**) Example of CD45^+^ cell proliferation in allogeneic grafts. Scale bar: 100 µm. (**C**) Quantification of CD3^+^ cell proliferation in autologous and allogeneic grafts. (**D** and **E**) Quantification of glucagon^+^ α (**D**) and C-peptide^+^ β (**E**) cell proliferation in autologous and allogeneic grafts. (**F**) Example of α and β cell proliferation in allogeneic grafts. Scale bar: 100 µm. (**G**) Example of CD45^+^ α and β cell proliferation in autologous grafts. *n* = 5 allogeneic mice were injected with additional in vitro activated polyclonal T cells (a hollow triangle represents the allogeneic mouse not injected with polyclonal T cells). Scale bar: 100 µm. Panel in **F** represents a lower-magnification view of the area shown in Figure 3F, visualized with different markers. Data shown as mean ± SEM; statistical analysis was performed with an unpaired 2-tailed *t* test for **A** and **C**–**E**. *P < 0.05. C-1, cohort 1; C-2, cohort 2.

We observed higher cell proliferation in allogeneic grafts compared with autologous grafts. CD45^+^ Ki67^+^ cells were significantly enriched in allogeneic grafts (Figure 6A), with some allogeneic grafts displaying proliferation preferentially among CD45^+^ cells, likely due to an active ongoing immune response (example in Figure 6B). The percentage of proliferating CD3^+^ T cells was also higher in allogeneic grafts (Figure 6C).

Surprisingly, glucagon^+^ and C-peptide^+^ cells were also more proliferative in allogeneic grafts compared with autologous grafts (Figure 6, D and E). In some allogeneic grafts, proliferation occurred preferentially in endocrine cells relative to CD45^+^ cells (example in Figure 6F). This suggests a possible regenerative capacity after destruction of SC-islet grafts, which might contribute to circulating C-peptide levels that remained high throughout the course of the study (Figure 2E), despite active ongoing destruction of allogeneic grafts. In contrast, autologous grafts showed more limited proliferation among both endocrine and immune cells (example in Figure 6G).

## Discussion

Here, we report a fully isogenic in vivo model whereby HIS mice were successfully grafted with autologous SC-islets. We used this in vivo model to study immune infiltration in autologous versus allogeneic SC-islet grafts by a fully reconstituted human immune system. Previous models assessed rejection of allogeneic grafts using human PBMCs or cord blood CD34^+^ cells (41). In the latter case, human CD34^+^ cells developed into T cells in the mouse thymus and were therefore not positively selected on human HLA, which constitutes a caveat (41). Deuse et al. used a HIS model including human thymic tissue to assess the escape of allogeneic hypoimmunogenic islets from rejection (42), without insights into the immune makeup of infiltrated grafts. Our fully isogenic model used human thymic tissue grafts that supported the development of HLA-A2/DR4/DQ8–restricted T cells, allowing the introduction of transgenic autoreactive TCRs to assess autoimmunity. We also report the successful reprogramming of fetal liver CD34^–^ cells into iPSCs with high efficiency, as previously described with other sources (43). Circulating C-peptide levels correlated with the efficiency of iPSC-to-β cell differentiation in vitro, irrespective of whether the grafts were autologous or allogeneic. However, these grafts also contained byproducts of incomplete β cell differentiation, such as enterochromaffin cells (identified as CD57^+^) and ductal cells (marked by CK19 expression), not typically observed in endogenous or transplanted allogeneic primary pancreatic islets. Although efforts in the field are ongoing to improve differentiation efficiency (2, 44), our findings underscore the importance of achieving high β cell frequency and function for SC-islets used in replacement therapies, in both autologous and allogeneic settings.

An unexpected finding was the substantial level of endocrine cell proliferation observed, particularly in allogeneic grafts. More Ki67^+^ α and β cells were observed in the presence of an allogeneic immune system, but whether this proliferation was promoted by immune cells is unclear. This cycling may nonetheless explain the fact that we had not observed a decrease in circulating C-peptide at the time we assessed infiltration. Interestingly, cycling α cells have also been suggested to serve as progenitors for new β cells via transdifferentiation in mice (45) and humans (46), although final proof using genetic lineage tracing has only been provided in mice.

Our results demonstrate immune infiltration and substantial damage in allogeneic islet grafts, with no clear signs of rejection in autologous grafts. Allorejection was mediated by immune cells, primarily found in clusters and containing an increased number of CD8^+^ T cells relative to autografts. CD68^+^ CD163^+^ macrophages were also enriched in allografts, where they might facilitate the profibrotic process, consistent with previous findings in allograft fibrosis (22). However, additional possible explanations for the unexpected increase of circulating C-peptide levels in mice implanted with allogeneic grafts include incomplete myeloid or NK cell reconstitution, as well as the fetal origin of the immune system.

Our observations demonstrate that T cells generated de novo in the human fetal thymic graft of HIS mice have the ability to infiltrate human SC-islet grafts. Previous studies have shown that human T cells generated in this manner have the capacity to spontaneously reject porcine islet xenografts and that T cells are critical for such rejection and responsible for recruiting human B cells and antigen-presenting cells (APCs) into the grafts (47). Interestingly, immune infiltration in autologous grafts was primarily concentrated around cyst-like structures formed by ductal cells, suggesting a possible recruitment of immune cells by CK19^+^ cells or local inflammation around these structures.

In a cohort of autologous mice, we adoptively transferred engineered IAR-TCR^+^ T cells, which did not appear to infiltrate the autologous SC-islet grafts, despite 2 clones being detected in the spleen 3 weeks after injection. Although there were few T cells in autologous grafts, we were not able to categorically identify them as our IAR-TCR^+^ T cells due to unreliable detection of GFP^+^ and NGFR^+^ cells by IMC. Notably, the injection of a large number of ex vivo activated autologous polyclonal T cells did not lead to any observed systemic adverse effects such as cytokine storm. Because we did not see any increase in infiltration with injection of IAR-TCR^+^ T cells, we were unable to evaluate any effect of SAgA treatment on insulitis in these mice. However, we observed phenotypic alterations on Clone 5 TCR^+^ CD4^+^ T cells after multiple treatments with T1D-SAgA (relative to control HAg-SAgA). A higher proportion of these human T cells lost IL-2Rα and/or gained PD-1 expression, which may indicate some degree of immunomodulation whereby these T cells reduced their proliferative potential while becoming more susceptible to inhibition or exhaustion via PD-L1.

Despite a substantial number of IAR-TCR^+^ T cells injected into each mouse, the lack of increased T cell infiltration of islet grafts relative to non-injected control mice raises several questions about factors that may limit autoimmune infiltration and the use of this model to recapitulate aspects of human T1D pathogenesis. An insufficient number of IAR-TCR^+^ T cells, including the use of a single CD8^+^ T cell clone, and a short observation period are possible contributors to the limited infiltration observed. After prolonged in ex vivo expansion and prior to injection, these T cells displayed an effector and terminally differentiated phenotype, which may reduce their long-term survival in vivo. The grafted SC-islets might also require an initial insult — such as ER stress or an inflammation-inducing event (14) — to promote immune infiltration, even in the presence of IAR-TCR^+^ T cells with multiple specificities. Excluding potential technical limitations, the results indicate that IAR-TCR^+^ T cells alone do not infiltrate the grafted islets but may require microenvironments resembling those of human islets. The lack of proper lymphatic formation, and therefore of lymph nodes draining the site of islet graft, in reconstituted NSG mice may also contribute to limited local engagement and stimulation of autoreactive IAR-TCR^+^ T cells. This HIS model does not support the testing of primary autologous islets, so it is difficult to conduct comparative studies to address whether SC-islets might be inherently less immunogenic, for example, through higher resistance to stress- or inflammation-induced alterations at the level of the immunopeptidome and their ability to present epitopes to T cells. Additionally, the unexpectedly high number of α cells observed — possibly related to a high fraction of C-peptide^+^ cells not expressing NKX6.1^+^ at the end of the differentiation (48) — surrounding β cells in vivo may help mask or shield some of the β cells.

Several studies have already indicated that T1D immune systems display abnormalities at the level of the hematopoietic compartment (49, 50), likely influenced by predisposing polymorphisms. The donor for our studies carried the high-risk HLA-DR4/DQ8 allele and had a very high non-HLA genetic risk 2 score based on 32 SNPs, exceeding the 99th percentile among ancestry-matched individuals, yet also carried the protective HLA-DQ6 allele, which may have contributed to tamping down the autoimmune T cell response. The mechanisms by which HLA-DQ6 protects from T1D are not well understood (51, 52), but these might include the development of regulatory T cells that could counteract the destructive effects of autoreactive T cells.

In this HIS model, both autologous and allogeneic grafts were infiltrated by human CD68^+^ CD163^–^ macrophages and CD14^+^ CD16^–^ monocytes, which can serve as APCs to potentially present peptides to antigen-specific T cells. Clone 5 T cells were the only IAR-TCR^+^ clone that consistently persisted long-term in vivo after adoptive transfer. Of note, the specific epitope (InsB_9-23_) for this clone is 100% homologous between mouse and human insulin, and the human APCs that present the abundant mouse version may provide homeostatic signals that improve their survival (53). This is in contrast to other T cell clones whose epitopes do not exist in native mouse tissues and were generally no longer detected 3 weeks later. Interestingly, delivery of InsB_9-23_ in SAgA form altered the response of Clone 5 T cells without affecting their relative frequency. Unlike other models featuring the transfer of human PBMCs, T cells in our model were centrally tolerized to many recipient antigens due to the presence of murine APCs in the human thymic graft (49). This likely contributed to a lack of signs of graft-versus-host disease throughout the course of our studies.

The model described above can serve as a valuable tool for evaluating the effects of therapies on human immune cell activation and graft infiltration and may help identify strategies to improve the success of SC-islet transplantation and β cell replacement therapies. A simpler version combining human immune systems and SC-islets that are HLA-mismatched can be used to test biologics targeting immune cells or engineered islets that have been rendered hypoimmune, for example. A more complex version, like the one presented here, has utility for evaluating the effects of antigen-specific therapies on human autoreactive T cells but requires human tissues with specific HLA haplotypes and IAR-TCRs. However, further improvement of the model is needed to assess the (auto)immune infiltration of autologous grafts, for the purposes of both T1D modeling and therapy evaluation. If possible, testing should be conducted in cohorts made from multiple donors for better generalizability (here, a single donor was used for all groups and cohorts). Future studies will build on this model by addressing the aforementioned limitations and refining the factors that promote or facilitate the infiltration of IAR-TCR^+^ T cells into autologous grafts.

## Methods

### Sex as a biological variable.

Both male and female NSG mice were used as recipients, with similar findings found for both sexes. After HIS reconstitution, mice were randomly assigned to the different groups.

### Mice.

NSG mice were purchased from The Jackson Laboratory, stock #005557. All mice were housed in a specific pathogen–free microisolator environment and used between 8 and 30 weeks of age.

### Human tissues.

Human fetal thymus and liver tissues of gestational age of 17–21 weeks were obtained from Advanced Bioscience Resource (now Cercle Allocation Services). Briefly, the connective and adipose tissue surrounding the thymus was removed, and the thymic tissue was cut into small fragments (~1 mm^3^). The liver was chopped into small pieces and digested with Liberase (Roche). CD34^+^ cells were then separated from CD34^–^ liver cells using the CD34 Microbead kit, following the manufacturer’s protocol (Miltenyi Biotec). Thymic fragments and CD34^+^ and CD34^–^ liver cells were cryopreserved and stored in liquid nitrogen.

### HLA typing and genetic risk score analysis.

CD34^–^ liver cells were HLA-typed by deep-sequencing at Histogenetics. The genotype of the fetal donor is shown in Supplemental Table 1, along with the non-HLA–related T1D genetic risk score. For the latter, SNP analysis was performed using the Illumina Infinium Global Screening Array at the Genomics Core Facility at the Icahn School of Medicine at Mount Sinai, generating 654,027 SNPs. Standard quality control filters were applied using PLINK v2.0.0 (https://www.cog-genomics.org/plink/2.0/). SNPs with genotype missingness greater than 5%, individuals with missing genotype rate greater than 5%, and SNPs deviating from Hardy-Weinberg equilibrium (HWE *P* < 1 × 10^–6^) were excluded. Ancestry was inferred based on principal component analysis on high-quality linkage disequilibrium-pruned SNPs overlapping with the 1000 Genomes Project reference panel. A random forest classifier trained on reference principal components was used to assign ancestry to study participants. Imputation was performed with Minimac4 using the TOPMed reference panel (54, 55). The T1D non-HLA genetic risk score, based on non-HLA SNP weights from the T1D genetic risk score 2 (56), was calculated from imputed data with imputation quality (*R*^2^) of 0.3 or higher. Post hoc ancestry adjustment was performed using the method described by Khan et al. (57). The adjusted genetic risk score was then compared with ancestry-matched individuals from 1000 Genomes to calculate ancestry-specific percentiles.

### Cell reprogramming to iPSC.

CD34^–^ fetal liver cells were reprogrammed to iPSCs using a nonintegrating Sendai virus method (CytoTune-iPS 2.0 Sendai Reprogramming kit, Thermo Fisher Scientific), with MOI 5:5:3 for KOS(Klf4–Oct3/4–Sox2)/c-Myc/Klf4 (58) following the manufacturer’s instructions; the remaining cells were stored. Small colonies with typical iPSC morphology started appearing after 12 days of transduction, and single clone picking was performed as previously described (18). After characterization, iPSCs were cultured on Matrigel-coated plates (Corning) in mTeSR Plus medium (Stemcell Technologies).

iPSCs for allogeneic SC-islets were reprogrammed from the skin fibroblasts of an adult donor with mismatched genotype (Supplemental Table 2).

### Differentiation of iPSCs to SC-islets.

Prior to induction of differentiation, all iPSC lines were maintained under feeder-free conditions in StemFlex medium (Thermo Fisher Scientific) at 37°C in a humidified incubator with 5% CO_2_ and routinely passaged every 3–5 days using TrypLE Express (Life Technologies). Directed differentiation into pancreatic lineages was performed using a previously optimized stepwise protocol (59), with the addition of aphidicolin to enhance both differentiation efficiency and transplantation outcomes (20). At the late stage (day 27) of differentiation, SC-islet-like clusters were enzymatically dissociated into single-cell suspensions for flow cytometric analysis of C-peptide and NKX6.1 expression (NovoCyte Penteon/Quanteon). After quality control assessment, approximately 100 day-27 SC-islet–like clusters, each containing around 2 × 10^4^ cells, were resuspended in 25 μL Matrigel (Corning) and prepared for kidney capsule transplantation.

### Generation and monitoring of HIS mice.

HIS mice were generated as described in previous studies (15, 16). Briefly, NSG mice were first thymectomized as described (60); they were then conditioned with sublethal (1.0 Gy) total body irradiation and received human CD34^+^ fetal liver cells (0.5 × 10^5^) intravenously and a fetal thymus tissue fragment (~1 mm^3^; under the right kidney capsule) from the same donor. In addition, either autologous or allogeneic SC-islets (100 day-27 islet-like clusters/mouse) were transplanted under the left kidney capsule between 4 and 10 weeks after humanization.

Human reconstitution in peripheral blood was monitored every 4–5 weeks, beginning 9–11 weeks after HIS reconstitution. Approximately 75 μL of whole blood was collected by tail vein puncture. PBMCs were isolated from peripheral blood by density gradient separation using Histopaque 1077 (Sigma-Aldrich) for flow cytometry analysis (Cytek 5-laser Aurora). Levels of human chimerism and T cells were monitored; 2 out of 28 mice with less than 5% T cells among human CD45^+^ cells, indicating poor thymic engraftment, were excluded from subsequent analyses.

### Monitoring of C-peptide levels.

Circulating human C-peptide was measured on 20 μL of plasma, isolated from whole blood, using the STELLUX Chemi Human C-peptide ELISA kit (Alpco). C-peptide levels were monitored every 3 weeks after islet transplantation.

### Lentiviral transduction of primary T cells.

The sequences of Clone 5, D222D, and R164 TCRs were provided by Bart Roep (Leiden University, Leiden, the Netherlands), Roberto Mallone (INSERM, Paris, France), and Helena Reijonen (City of Hope, Duarte, California, USA), respectively. The sequences of clones A1.9 and A3.10 TCRs were inferred from the CDR3α/β sequences and VJα and VDJβ information published by Pathiraja et al. (25). The D222D, Clone 5, A3.10, R164, and A1.9 TCRs were subcloned into a pRRL-MSCV-TCR-F2A-reporter second generation lentiviral backbone. For Clone 5, the final plasmid construct contained the following (in this order): TCRα sequence, P2A site, TCRβ sequence, F2A site, and GFP sequence; for the other clones, the final plasmid construct contained (in this order): TCRβ sequence, P2A site, TCRα sequence, F2A site, and reporter (as indicated in Table 1). Viruses were produced in HEK293 FT cells by standard procedures (61). Splenocytes from a donor mouse reconstituted with HIS were activated by ImmunoCult Human CD3/CD28/CD2 T Cell Activator (Stemcell Technologies) and cultured in X-VIVO 15 (Lonza Bioscience) supplemented with IL-2, IL-7, and IL-15 (PeproTech). After 3 days, cells were transduced overnight with viruses using magnetofection (Viromag, OZ Biosciences), either with (cohort 1) or without (cohort 2) CRISPR KO of the endogenous TCRα and TCRβ chains, as described previously (62). Cells were expanded in regular 24-well plates (cohort 1) or G-Rex 24-well plate (cohort 2, Wilson Wolf) in X-VIVO 15 supplemented with IL-2, IL-7, and IL-15. In cohort 2, X-VIVO 15 was further supplemented with phytohemagglutinin and irradiated PBMCs. Adoptively transferred mice were injected with 0.2 × 10^6^ to 0.5 × 10^6^ TCR^+^ reporter^+^ per clone per mouse; mice received 6 × 10^6^ (cohort 1) and 18 × 10^6^ (cohort 2) total activated T cells (IAR-TCR^+^ and polyclonal). In 5 out of 6 allogeneic mice, in vitro–activated polyclonal T cells autologous to the HIS (3 × 10^6^/mouse) were injected to complement endogenous T cells.

### SAgA production, in vitro validation, and treatment.

SAgAs were generated as follows. Briefly, approximately 6 InsB_9-23_ peptides were conjugated to approximately 22 kDa hyaluronic acid (HA) backbone via copper-catalyzed azide–alkyne cycloaddition chemistry to form InsB_9-23_-SAgA (31). HAg-SAgA was produced using reductive amination chemistry to chemically link approximately 9 HAg_306-318_ peptides to approximately 16 kDa HA. Lastly, Proinsulin-SAgA was synthesized via reductive amination chemistry conjugating approximately 3 proinsulin(F25D) molecules to approximately 16 kDa HA (29, 30). Confirmation of conjugated peptide/protein to HA was performed using proton nuclear magnetic resonance and size exclusion chromatography using ultraviolet light and refractive index detectors. All SAgAs were biophysically characterized using dynamic light scattering and circular dichroism. The production and characterization of these SAgAs are reported in more detail in more detail elsewhere (30, 31). For in vitro validation of InsB_9-23_-SAgA, Clone 5–transduced primary T cells were incubated for 3 days with splenocytes and HLA-DQ8-transgenic K562 (gifted by Maki Nakayama).

Starting at 9 weeks after islet graft transplantation, 2 subgroups of mice (*n* = 5/group) were injected subcutaneously at the neck fold twice a week with either 2.5 nmol T1D-SAgA (mixture of InsB_9-23_-SAgA and Proinsulin-SAgA) or 2.5 nmol HAg-SAgA, for a total of 7 injections/mouse.

### SC-islet graft processing, tissue staining, and imaging mass cytometry analysis.

SC-islet grafts were harvested from mice 14–18 weeks after transplantation. For experiments with adoptive transfer of T cells, grafts were harvested 3 weeks after injection. Grafts were fixed in formalin 10% and then transferred to ethanol for histological and IMC analysis.

IMC was performed on sections of FFPE samples, as previously reported (21). Briefly, tissue sections were cut at 6 μm and mounted on slides, which were then deparaffinized in 2× 20-minute washes of xylene and then gradually rehydrated by sequential washes from 100% through 70% ethanol. Slides were then washed briefly in PBS and transferred to Tris/EDTA (10 mM Tris, 1 mM EDTA, pH 9.2) buffer for antigen retrieval in a decloaking chamber (Biocare Medical) at 95°C for 30 minutes before being cooled at room temperature for 1 hour. Slides were then blocked in 3% BSA in PBS for 1 hour at room temperature and stained with approximately 100 μL/slide of the antibody cocktail overnight at 4°C in a humidified chamber. The next day, slides were stained for DNA by incubating for 30 minutes at room temperature with 1.25 μM Cell-ID Intercalator-Ir (Standard BioTools). Antibody and DNA-staining cocktails were diluted in 0.5% BSA in PBS. Batches of slides were then washed 1 time in an excess (~200 mL) of PBS and then twice in ultrapure 18.2 MΩ water, before being air dried and acquired on the IMC (Standard BioTools, Hyperion Imaging System) according to Standard BioTools’ standard operating procedures, with a 200 Hz laser frequency and a 1 μm step increment. The final panel consisted of 37 antibodies and the addition of antibodies targeting NGFR and GFP (Supplemental Table 3).

Images were analyzed using Halo software (Indica Labs, version 4.1); markers used are indicated in the figures. A random forest algorithm was trained to identify areas containing human cells in the images (islet graft, glucagon^+^ and C-peptide^+^ cells, and surrounding fibrotic tissue, pan-Keratin^+^ cells) and was then used to exclude the mouse kidney and cyst areas (HLA-ABC^+^ CK19^+^ cells) from the analysis. Images were used for training the algorithms to detect HLA-ABC^+^ CD45^+^ immune cells, and within this population, T cells (CD3^+^ CD4^+^ and CD3^+^ CD8^+^), monocytes (CD14^+^ CD16^–^ CD68^–^, CD14^+^ CD16^+^ CD68^–^, and CD14^–^ CD16^+^ CD68^–^), macrophages (CD68^+^ CD163^+^ and CD68^+^ CD163^–^), B cells (CD20^+^), and NK cells (CD3^–^ CD56^+^). The Ki67 marker was used to identify proliferating human cells. Granzyme B and FoxP3 markers were used to identify cells with expected cytotoxic and regulatory functions, respectively. The endocrine cells forming the grafts were characterized as follows: HLA-ABC^+^ C-peptide^+^ β cells, HLA-ABC^+^ C-peptide^+^ NKX6.1^+^ β cells, HLA-ABC^+^ glucagon^+^ C-peptide^–^ α cells, HLA-ABC^+^ SST^+^ δ cells, and HLA-ABC^+^ Ghrelin^+^ ε cells. Finally, density spatial analysis was used to count the number of HLA-ABC^+^ CD45^+^ immune cells within 100 μm of radius. HLA-ABC signal intensity on cells of islet grafts was quantified on a per-cell basis. HLA-ABC signal was classified into 3 arbitrary but internally consistent categories (weak, moderate, strong) using fixed intensity thresholds defined in Halo and applied consistently across all samples. The distribution of cells across these categories was compared between grafts.

### Spleen processing and flow cytometry analysis.

Spleens were crushed to generate a single-cell suspension after red blood cell lysis. Around 3 × 10^6^ to 5 × 10^6^ splenocytes were stained for flow cytometry analysis using the antibodies listed in Supplemental Table 4.

Flow cytometry data were acquired in the Columbia Center for Translational Immunology Flow Core. Data were collected using a Cytek 5-laser Aurora cytometer and analyzed using FlowJo (BD Biosciences). For evaluation of Clone 5 phenotype in the spleen, only samples with at least 20 GFP^+^ events were included in the analysis.

### scRNA-Seq analysis.

Raw FASTQ files for published scRNA-Seq data of in vitro SC-islet differentiations were downloaded from NCBI’s Gene Expression Omnibus (GEO) using the NCBI Sequencing Read Archive (SRA) toolkit (https://github.com/ncbi/sra-tools). Aligned raw counts were generated using the 10x Genomics Cell Ranger pipeline. Preprocessing and quality control filtering were performed using the standard Seurat workflow. Integration and batch effect removal was performed using the Harmony algorithm, implemented in Seurat v5. Gene expression was visualized using the VlnPlot() and FeaturePlot() functions.

### Statistics.

GraphPad Prism 10.4.1 was used to perform statistical analyses. One-way ANOVA followed by Tukey’s post hoc test for multiple comparisons, paired or unpaired 2-tailed *t* tests, and/or a mixed-effects model were performed as indicated in legends. A *P* value less than 0.05 was considered significant.

### Study approval.

Protocols involving the use of discarded human tissues and animals were approved by the Columbia University Medical Center IRB and the IACUC, respectively, and all experiments were performed in accordance with these protocols.

### Data availability.

The original contributions presented in the study are included in the article or supplemental material. Values for all data points in graphs are reported in the Supporting Data Values file. Inquiries can be directed to the corresponding author. Values for all data points in the graphs are reported in the Supporting Data Values file.

## Author contributions

CBG, GZ, KHK, CB, DE, MS, and RJC designed research studies. CBG, GZ, QD, DT, DL, GMD, SAB, XD, KDA, and RFF conducted experiments. CBG, GZ, QD, DT, and DL acquired data. CBG and GZ analyzed data. MI, EESC, JRM, and YL performed computational analyses. CBG and RJC wrote the manuscript with input from all authors. RJC and MS supervised the project. All authors have approved the final version of the manuscript.

## Conflict of interest

JRM is an inventor on a patent and patent application relating to stem cell–derived pancreatic islets, US 10,030,229 “SC-β cells and compositions and methods for generating the same” and US application PCT/US2019/032643 “Methods and Compositions for Generating Cells of Endodermal Lineage and Beta Cells and Uses Thereof.” JRM was recently employed at and has stock in Sana Biotechnology.

## Funding support

This work is in part the result of NIH funding, and as such, is subject to the NIH Public Access Policy. Through acceptance of this federal funding, the NIH has been given a right to make the work publicly available in PubMed Central.

- Breakthrough T1D award 2-SRA-2022-1220-S-B to RJC and MS.
- NIH grants R01AI142428 to RJC, U01DK123559 to MS, and R01DK127778 and P30DK026687 to DE for developed resources.
- Berrie postdoctoral fellowship in diabetes research and Berrie-Concordia scholarship to CBG.
- American Diabetes Association Postdoctoral fellowship 1-25-PDF-01 to GZ.
- Rita Levi-Montalcini postdoctoral fellowship in Regenerative Medicine to MI.
- NIH grants T32DK007120 to MI and R01DK114233, R01DK127497, R01DK138469, and UG3 DK142188 to JRM.
- Human Pancreas Analysis Program (HPAP-RRID:SCR_016202), a Human Islet Research Network (RRID:SCR_014393) consortium supported by National Institute of Diabetes and Digestive and Kidney Diseases grants UC4DK112217, U01DK123594, UC4DK112232, and U01DK123716, to KHK.
- Breakthrough T1D awards 3-SRA-2023-1295-S-B, 3-SRA-2024-1555-S-B, and 2-SRA-2023-1314-S-B to JRM.
- Edward Mallinckrodt Jr. Foundation to JRM.
- Anita Palmer Corbin Trust to JRM.
- NIH grants P30DK063608 and P30CA013696 to the Flow Cytometry Core, which provided resources.

## Supplementary Material

Supplemental data

Supporting data values

## Figures and Tables

**Table 1:**
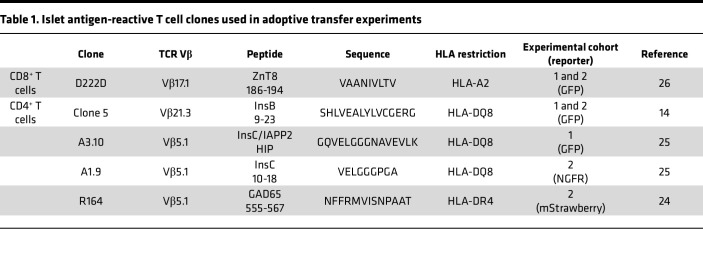
Islet antigen-reactive T cell clones used in adoptive transfer experiments

## Version 1 06/08/2026

Electronic publication
