# Supplementary material for: Modeling immune responses to autologous and allogeneic human stem cell–derived islet grafts in vivo
Source: JCI Insight. 2026 Jun 8;11(11):e200738. doi: 10.1172/jci.insight.200738 (PMC13313504; doi:10.1172/jci.insight.200738)
Supplement: Supplemental data [file jciinsight-11-200738-s287.pdf]

Supplemental Material

Modeling immune responses to autologous and allogeneic human stem cell-derived islet grafts in vivo

Camillo Bechi Genzano, Giorgia Zanetti, Qian Du, Daniel Traum, Deeksha Lahori, Grant M Downes, Sakshi A Bhatele, Xiaolan Ding, Kyle D Apley, Rebuma Firdessa Fite, Matthew Ishahak, Enrique Eduardo Sanchez-Castro, Jeffrey R Millman, Yiming Luo, Klaus H Kaestner, Cory Berkland, Dieter Egli, Megan Sykes, Remi J Creusot

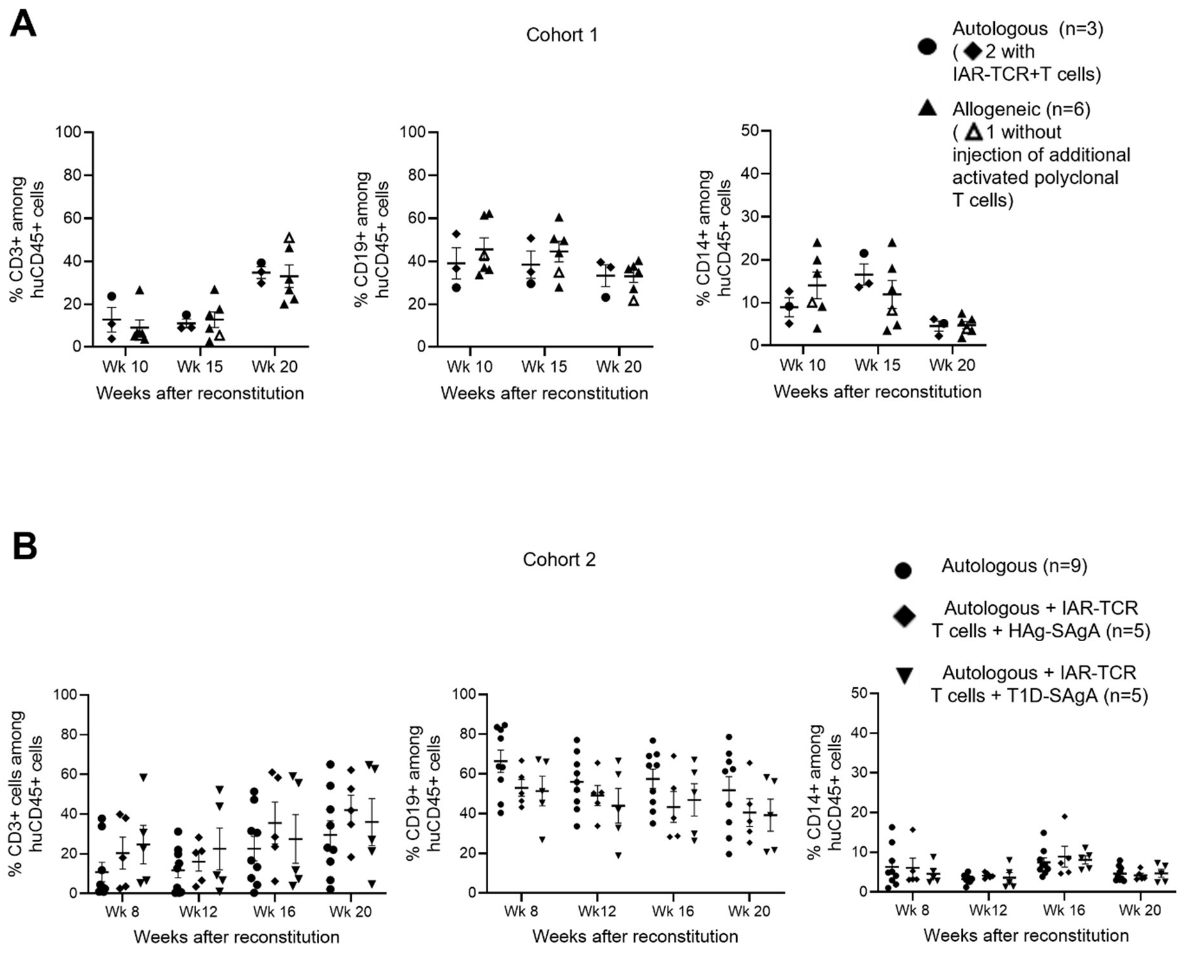

Figure S1. (A,B) Subpopulations of human immune system (CD3⁺ T cells, CD19⁺ B cells, CD14⁺ monocytes) in (A) cohort 1 and (B) cohort 2. In panels A and B, data show the mean ± SEM.

IAR-TCR, Islet antigen-reactive-T cell receptor; T1D-SAgA, Type 1 diabetes-Soluble Antigen Array; HAg-SAgA, Hemagglutinin-Soluble Antigen Array.

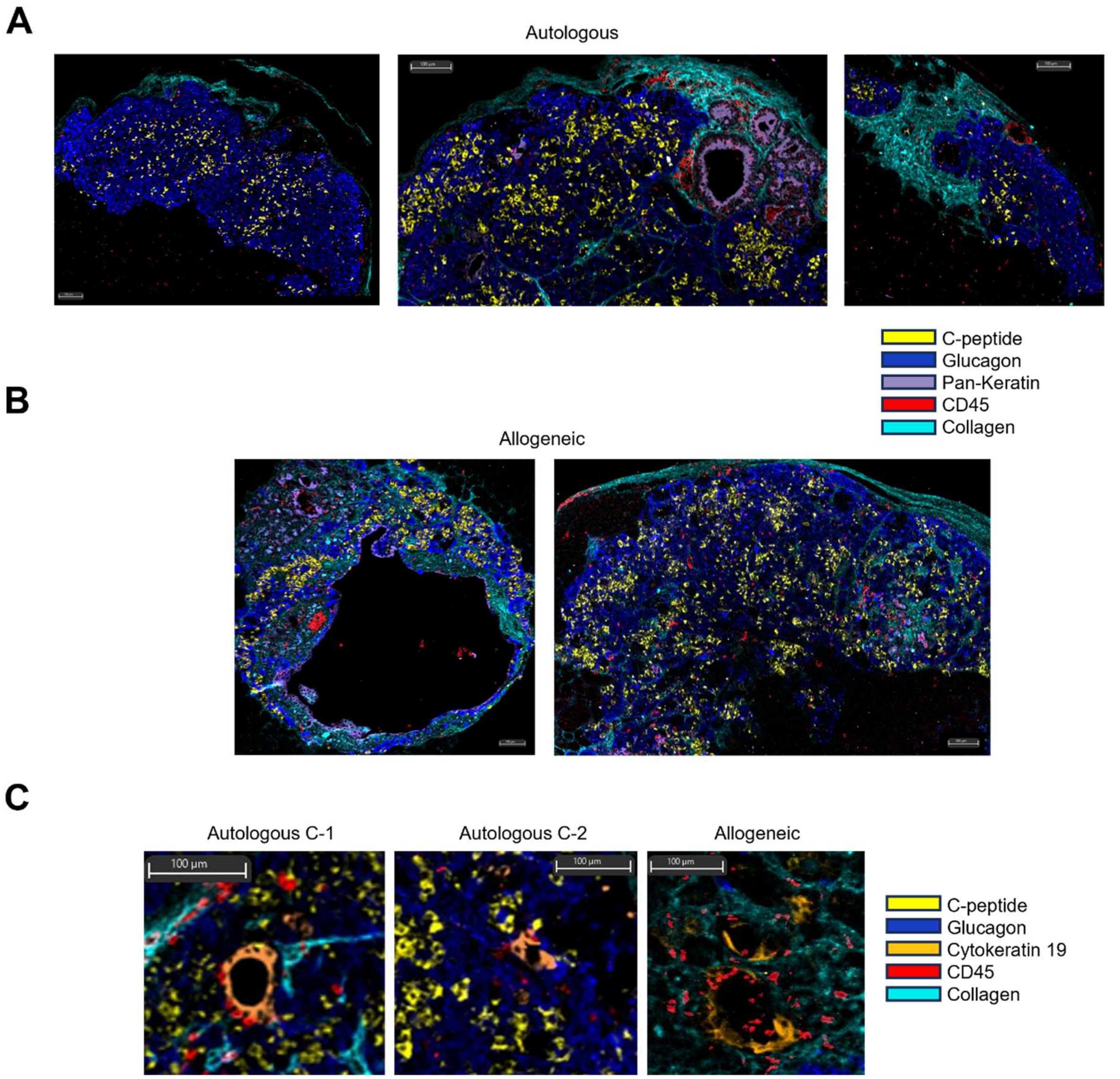

Figure S2. (A,B) Autologous (A) and allogeneic (B) SC-islet graft human immune infiltration. **(C)** Infiltration of cyst-like structures in autologous and allogeneic grafts. Markers used are indicated in the figure. The leftmost and the rightmost panel in (C) represent a magnified view of the regions shown in Figure 5D and S2B (left panel), respectively, visualized with different markers.

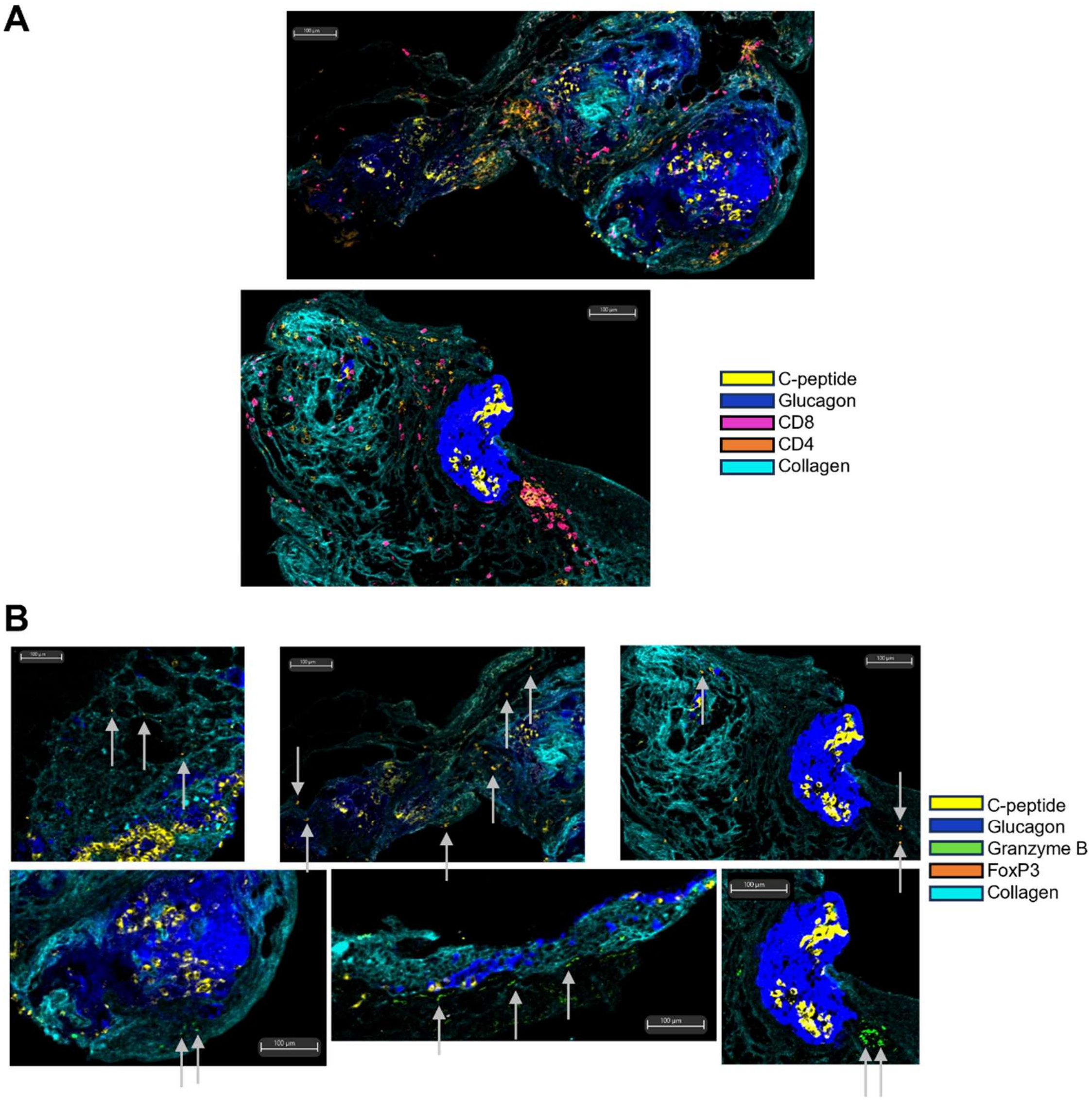

Figure S3. **(A)** T cell subpopulations in allogeneic grafts. **(B)** FoxP3⁺ and Granzyme B⁺ T cells in allogeneic grafts. Markers used are indicated in the figure. The upper panel in (A) and the leftmost-lower panel in (B) represent the same region shown in Figure 6B, visualized with different markers. The lower panel in (A) and the rightmost panel in (B) represent the same region shown in Figure 3B, visualized with different markers.

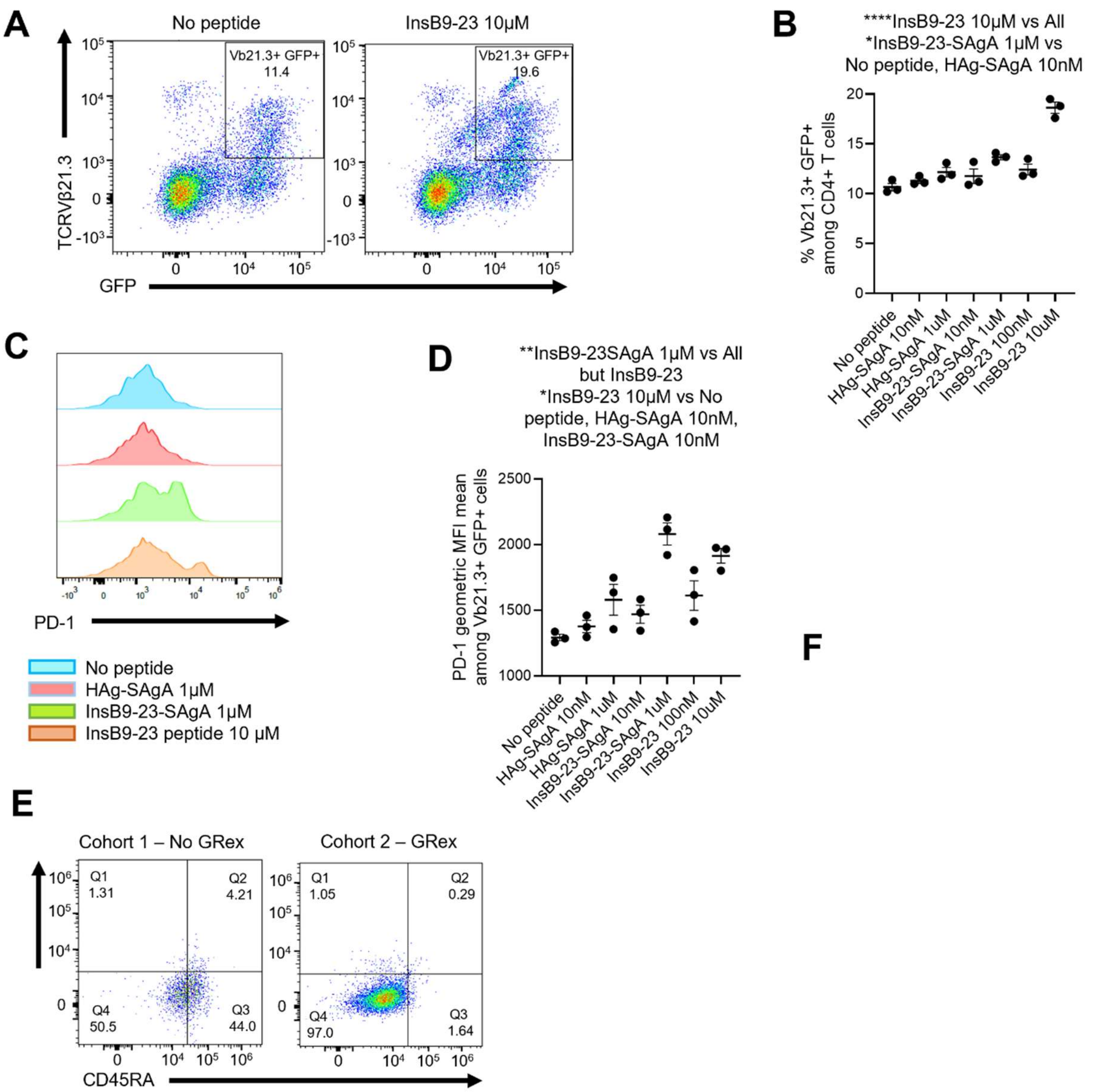

Figure S4. **(A-D)** Frequency (A,B) and activation marker PD-1 levels (C,D) of Vβ21.3⁺ GFP⁺ cells after three-day co-culture with K562-DQ8 cells and indicated compounds. **(E)** Phenotype of CD4⁺ T cells transduced with Clone 5 TCR-encoding lentivirus long term after in vitro activation; T cell expansion performed in regular plates/flasks (Cohort 1) or with G-Rex plates (Cohort 2). Data show the mean ± SEM; statistical analysis was performed using one-way ANOVA/Tukey for panels B and D. **(F)** Quantification of HLA-ABC signal intensity in cells of islet grafts.

InsB9-23-SAgA, InsulinB9-23-Soluble Antigen Array; HAg-SAgA, Hemagglutinin-Soluble Antigen Array.

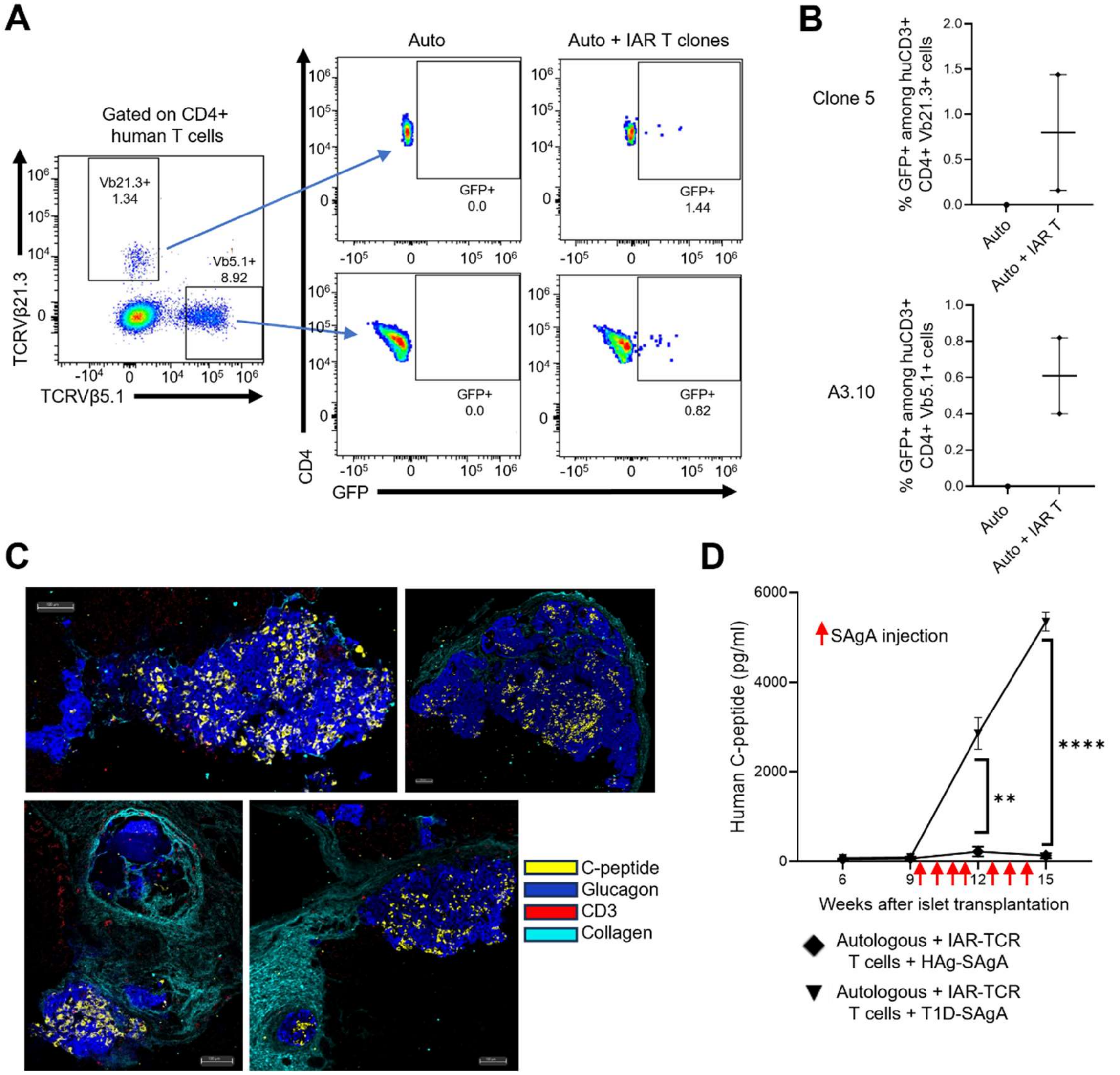

Figure S5. **(A,B)** Cohort 1: Clone 5 (Vβ21.3$^+$ GFP$^+$) and A3.10 (Vβ5.1$^+$ GFP$^+$) CD4$^+$ T cells in the spleen three weeks after adoptive transfer. **(C)** Autologous grafts injected with IAR-TCR$^+$ T cells and islet antigen-SAgAs. **(D)** C-peptide levels of mice transplanted with autologous grafts and injected with IAR T cells, then with InsB$_{9\text{-}23}$-SAgA and Proinsulin(F25D)-SAgA. Each red arrow indicates one weekly injection. Data show the mean ± SEM; statistical analysis was performed using a mixed effects model with Geisser-Greenhouse correction and Sidak for panel D.

IAR-TCR, Islet antigen-reactive-T cell receptor; T1D-SAgA, Type 1 diabetes-Soluble Antigen Array; HAg-SAgA, Hemagglutinin-Soluble Antigen Array.

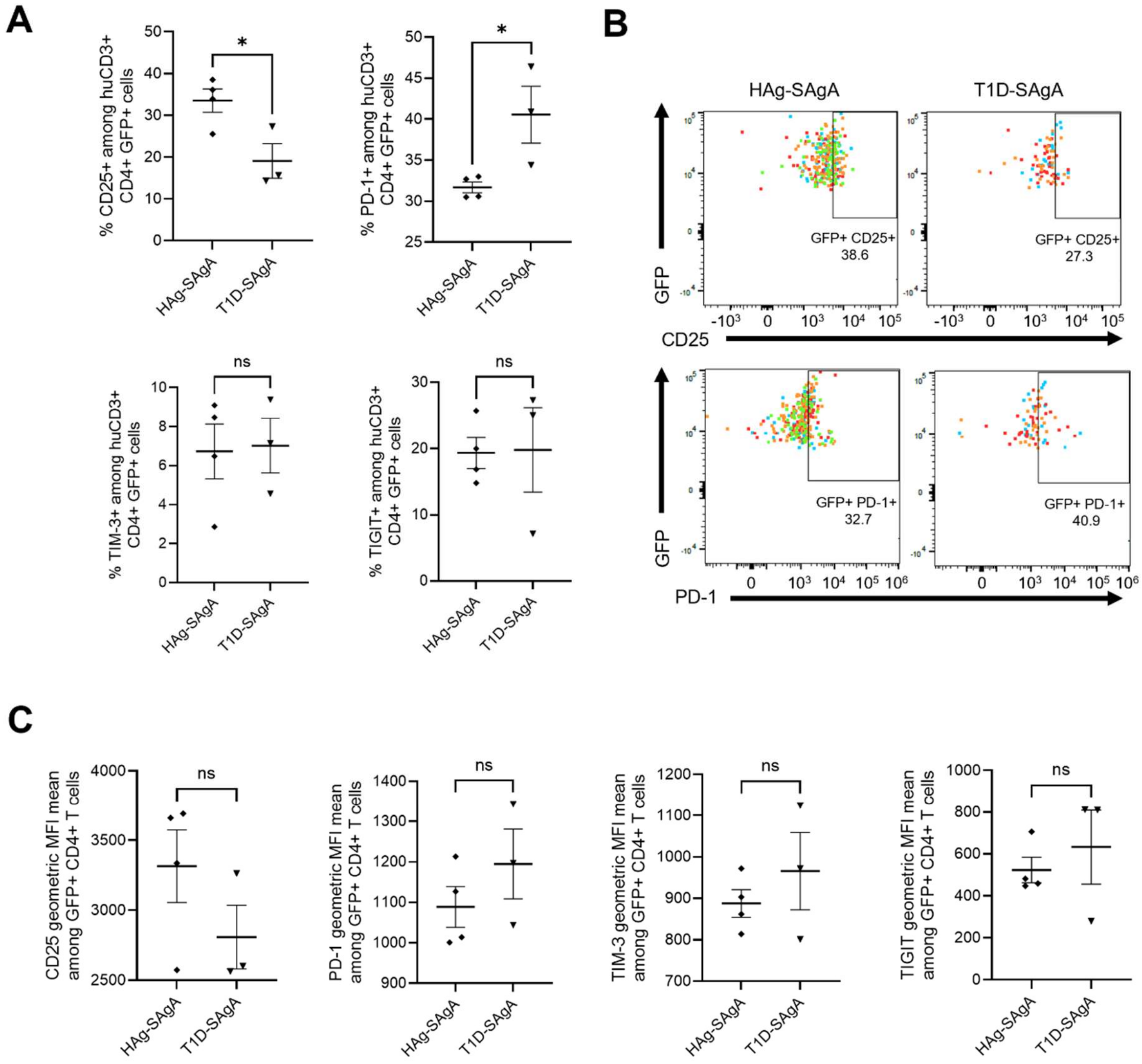

Figure S6. Phenotypic profile of adoptively transferred Clone 5 CD4^{+} T cells in spleen three weeks after injection. **(A)** Comparison of downstream markers CD25, PD-1, Tim3, Tigit between mice treated with HAg-SAgA or T1D-SAgA. **(B)** Concatenated plots of 4 mice treated with HAg-SAgA and 3 mice treated with T1D-SAgA. **(C)** Comparison of downstream markers CD25, PD-1, Tim3, Tigit MFI between the two groups. Data show the mean ± SEM; statistical analysis was performed using unpaired 2-tailed T test for panels A and C (* $P < 0.05$).

T1D-SAgA, Type 1 diabetes-Soluble Antigen Array; HAg-SAgA, Hemagglutinin-Soluble Antigen Array.

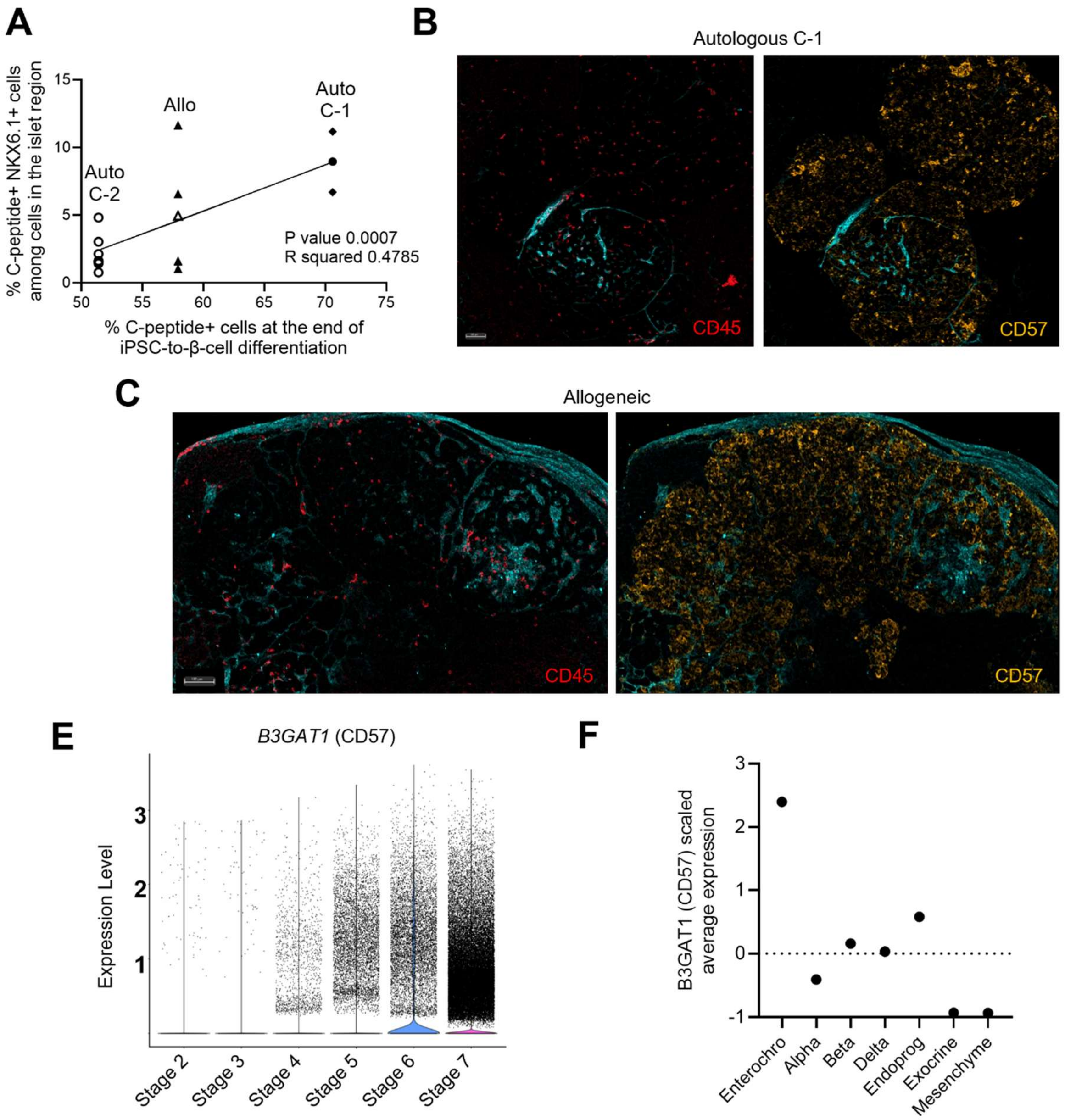

Figure S7. **(A)** Correlation between % C-peptide⁺ cells in islet pre-transplant and C-peptide⁺ NKX6.1⁺ cells in the graft. Each symbol represents a mouse. **(B,C)** Representative plot of CD57 expression in autologous (B) and allogeneic (C) grafts, compared to CD45. **(D)** Violin plot of CD57 gene expression demonstrating increased expression during in vitro differentiation of SC-islets and **(E)** Scaled average expression of *B3GAT1* (CD57) among SC-islet subpopulations, both from previously published scRNAseq data. The panel in (B) represents the same region shown in Figure 5D, visualized with different markers. The panel in (C) represents the same region shown in Figure S2B (right panel), visualized with different

markers. Statistical analysis was performed using a simple linear regression for panel A. In panel B, data shows the mean ± SEM; one-way ANOVA/Tukey was used for panel B. Enterochro, enterochromaffin; Endoprog, endocrine progenitors.

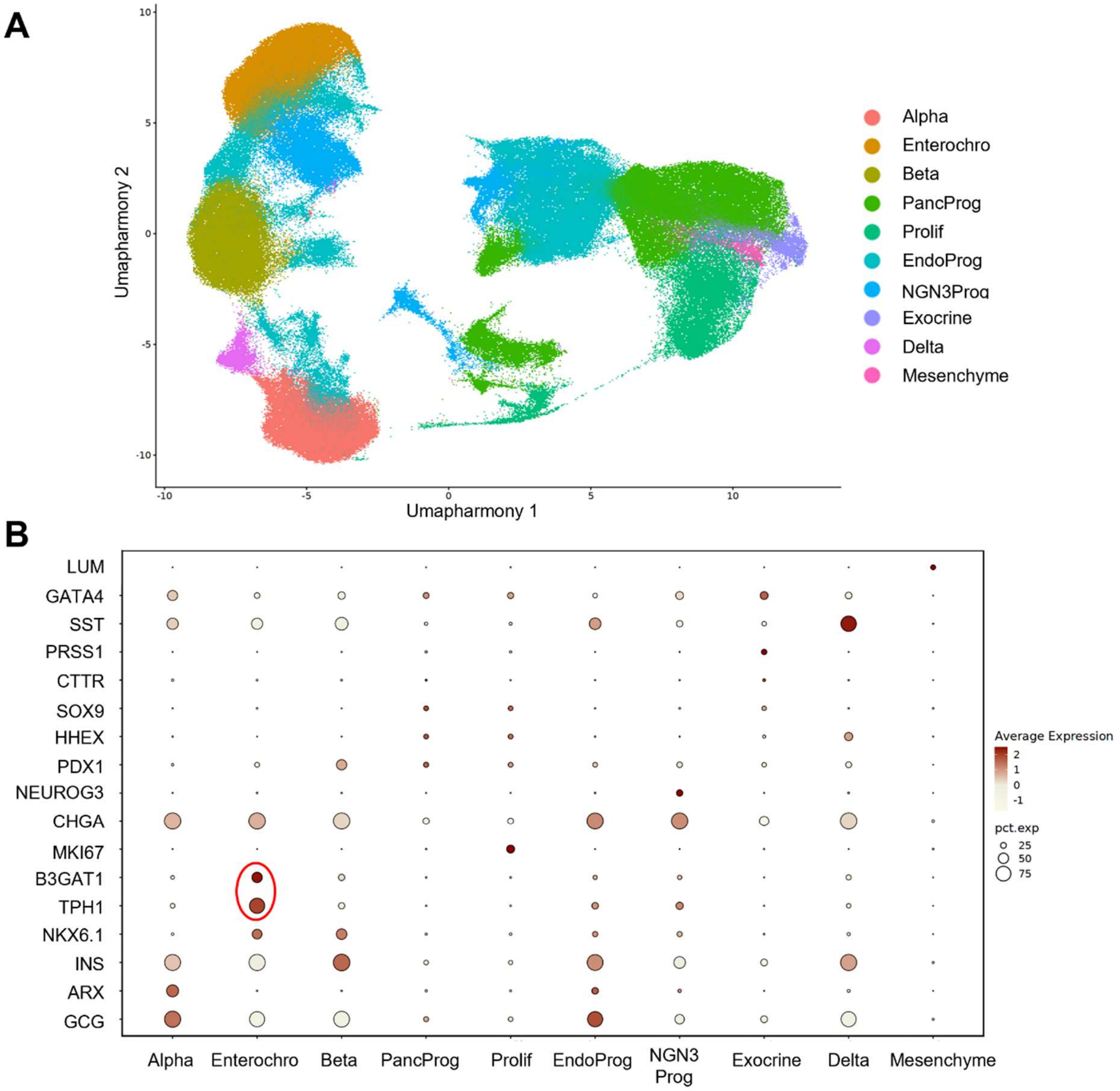

Figure S8. **(A)** UMAP and **(B)** gene expression profiles from multiple previously published scRNAseq data on SC-islets, as described in the main text. As highlighted in panel B, *B3GAT1* (CD57) is highly expressed in *TPH1* (tryptophan hydroxylase 1) positive cells, a marker of enterochromaffin cells.

Enterochro, enterochromaffin; PancProg, pancreatic progenitors; Prolif, proliferative; Endoprog, endocrine progenitors; NGN3 Prog, neurogenin progenitors.

	Gene	Allele 1	Allele 2
HLA	HLA A	02:01:01	29:02:01
	HLA B	07:02:01	27:05:02
	HLA C	02:02:02	07:02:01
	HLA DQB1	03:02:01	06:02:01
	HLA DRB1	04:04:01	15:01:01
Non-HLA	**SNP**	**Gene**	**Beta**
	rs3842753_G	INS	0.83
	rs2476601_A	PTPN22	0.64
	rs2289702_C	CTSH	0.28
	rs653178_C	ATXN2	0.26
	rs4948088_C	COBL	0.26
	rs9924471_A	IL27	0.22
	rs4759229_A	ERBB3	0.22
	rs1893217_G	PTPN2	0.19
	rs72928038_A	BACH2	0.18
	rs60888743_A	RNLS	0.18
	rs11170466_T	ITGB7	0.17
	rs9981624_C	UBASH3A	0.17
	rs9388489_A	CENPW	0.16
	rs5763779_A	HORMAD2	0.15
	rs425105_T	PRKD2	0.15
	rs72727394_T	RASGRP1	0.14
	rs17388568_A	ADAD1	0.12
	rs1615504_T	CD226	0.12
	rs6476839_T	COBL	0.11
	rs9585056_C	IRF7	0.11
	rs229541_A	C1QTNF6	0.1
	rs2281808_C	SIRPG	0.1
	rs1738074_T	TAGAP	-0.08
	rs56994090_C	MEG3	-0.13
	rs10492166_A	CLEC1	-0.14
	rs3024505_A	PTPN22	-0.15
	rs2111485_A	IFIH1	-0.16
	rs3087243_A	CTLA4	-0.17
	rs12708716_G	CLEC16A	-0.19
	rs144309607_T	TYK2	-0.4
	rs61839660_T	IL2RA	-0.48
	rs41295121_T	IL2RA	-0.71

Table S1. HLA genotype and non-HLA related SNPs of the autologous donor. Non-HLA related SNPs were used to quantify the T1D genetic risk score. The Beta coefficient indicates the weight of a specific SNP on the risk score.

	Gene	Allele
HLA	HLA A	03 and 11
	HLA B	35 and 56
	HLA DQA1	05:01 and 01:03
	HLA DQB1	03:01 and 06:03
	HLA DR1	11:01 and 13:01

Table S2. Genotype of allogeneic donor.

Isotope	Mass	Target	RRID
Yb	89	Collagen	AB_92249
In	115	CK19	AB_439773
Pr	141	HLA-ABC	AB_2739161
Nd	142	CD57	AB_2868399
Nd	143	CD31	LS Bio, LS-B15507
Nd	144	CD14	AB_2924314
Nd	145	C-peptide	AB_558517
Nd	146	Nestin	AB_2889996
Sm	147	Glucagon	AB_2629431
Nd	148	pan-Keratin	AB_439773
Sm	149	CD11b	AB_2891189
Nd	150	CD44	AB_2827882
Eu	151	PDX-1	AB_355257
Sm	152	CD45	AB_2909538
Eu	153	CD56	AB_2149421
Sm	154	beta-actin	AB_2938624
Gd	155	Foxp3	eBioscience, 14-477-82
Gd	156	CD4	AB_2811051
Gd	158	NKX6.1	AB_10673664
Tb	159	CD68	AB_2810859
Gd	160	SST	AB_831726
Dy	161	CD20	AB_2811016
Dy	162	CD8	AB_2909535
Dy	164	CD99	AB_2076301
Ho	165	CA2	AB_2612117
Er	166	pNFkB	AB_2847867
Er	167	Granzyme B	AB_2811057
Er	168	Ki67	AB_2811061
Tm	169	CD16	AB_2877105
Er	170	CD3	AB_2811048
Yb	171	IAPP	SantaCruz, sc-377530
Yb	172	CHGA	AB_2738844
Yb	173	CD45RO	AB_2811052
Yb	174	CD163	AB_2074540
Lu	175	PP	AB_1524152
Yb	176	Ghrelin	AB_2232479
Bi	209	HLA-DR	AB_445401
Sm	154	NGFR	AB_2564818
Er	166	GFP	Abcam, ab220802

Table S3. List of antibodies used in IMC experiments.

Antibody	Fluorophore	Clone	RRID	Experiment
C-peptide	Rat	GN-ID4	AB_2255626	1
Anti-Rat IgG	AlexaFluor488	N/A	AB_2535794	1
NKX6.1	Mouse	F55A10	AB_532378	1
Anti-mouse IgG	AlexaFluor647	N/A	AB_162542	1
Zombie UV	N/A	N/A	Biolegend 423108	2
CCR7	BUV395	3D12	AB_2937088	2
CD3	BUV737	UCHT1	AB_2895919	2
Ter-119	BUV805	TER-119	AB_2896157	2
CD11c	BV421	3.9	AB_11203895	2
CD4	Pacific Blue	OKT4	AB_571953	2-4
CD14	BV605	M5E2	AB_2563798	2
CD25	BV650	BC96	AB_2563807	2-5
CD45	BV711	HI30	AB_2563465	2
NGFR	PerCP/Cy5.5	ME20.4	AB_11204075	2
TCR Vb21	PE	REA894	AB_2726796	2
CD127	PE/Cy7	A019D5	AB_10899414	2
TCR Vβ5.1	APC	LC4	AB_2733454	2-3
CD8	Alexa Fluor 700	HIT8a	AB_528884	2
mCD45	APC/Cy7	30-F11	AB_312980	2-5
CD19	APC/Fire 810	HIB19	AB_2860770	2
CD45RA	BV510	HI100	AB_2561947	2-5
TCR Vβ21.3	APC	REA894	AB_2726797	3-4-5
TCRαβ	APC	IP26	AB_10612747	3
NGFR	FITC	ME20.4	AB_2282828	3
TCR Vβ17	PE/Vio 770	REA915	AB_2726961	3-5
CD127	APC/Fire 810	A019D5	AB_2904371	4-5
Tigit	BV421	A15153G	AB_2632924	5
CD69	PE/Cy7	FN50	AB_314846	4-5
CD45	BUV395	HI30	AB_2920949	4-5
CD3	BUV615	OKT3	AB_2925402	4-5
CD25	BUV737	BC96	AB_2895955	4-5
CD8	BUV805	RPA-T8	AB_2896080	4-5
PD-1	BV711	EH12.2H7	AB_11218612	4-5
NGFR	Pacific Blue	ME20.4	AB_2894480	5
TIM-3	BV650	F38-2E2	AB_2565828	5
CD4	Spark Blue 574	SK3	AB_2910397	5
CD69	BV786	FN50	AB_2925732	5
Ter-119	PerCP/Cy5.5	TER-119	AB_893636	5
CD19	RB780	SJ25C1	AB_3684459	5
CCR7	PE/Cy5.5	3D12	AB_2815128	5

Table S4. List of antibodies used in flow cytometry experiments. Experiments: (1) in vitro β cell differentiation, (2) chimerism check, (3) T cell transduction, (4) in vitro T cell validation, (5) spleen analysis.
